# Proceedings From the First Asia-Oceania Research Organisation on Genital Infections and Neoplasia (AOGIN) Meeting

**DOI:** 10.1155/IDOG/2006/59089

**Published:** 2006-11-09

**Authors:** Edited by Sebastian Faro

**Affiliations:** Department of Obstetrics and Gynecology, The Woman's Hospital of Texas, Houston Texas 77054, USA

## Abstract

The First Asia-Oceania Research Organisation on Genital Infections and Neoplasia (AOGIN) Meeting was held in Kota Kinabalu, Malaysia, in July 2005. The conference covered regional issues relating to infection with the human papillomavirus—epidemiology, virology, and immunology, testing, screening, and prevention strategies—as well as cervical cancer screening and its management.

## INTRODUCTION TO AOGIN, THE ASIA OCEANIA RESEARCH ORGANISATION ON GENITAL INFECTIONS AND NEOPLASIA

### Suzanne Garland

#### The need

Cancer prevention and control are among the most important public health challenges facing the world today.

Worldwide, carcinoma of the uterine cervix is the second most common cancer in women. Incident cases are estimated to be approximately 470 600 per annum, with almost 80% occurring in the developing world.

#### The organisation

The AOGIN concept was based upon an expert multidisciplinary group developed overseas—the European Research Organisation on Genital Infection and Neoplasia (EUROGIN).

This organisation crosses professional boundaries, uniting gynaecology, sexual health, dermatology, epidemiology/public health, pathology, biology, oncology, and basic science.

#### Objectives and activity

AOGIN aims are to promote and develop research, training, screening, prevention, and information concerning genital infections, precancers, and cancers in women.

New breakthroughs in diagnostics, treatment, and prevention will provide both opportunities and challenges in upcoming years. AOGIN activity should assist decision-making and planning so that the most cost-effective gains can be made for each nation.

The work will focus on

1. collaboration and research,
2. scientific exchanges, education and training,
3. information provision,
4. surveys and audits.

#### First meeting

The first AOGIN Meeting was held in Kota Kinabalu, Malaysia, in July 2005. It was coordinated by an executive committee that included leading physicians from Australia, Japan, Korea, Singapore, India, and China.

The meeting was generously supported by a number of pharmaceutical and diagnostic companies, and received helpful input from professional societies. Delegates from 23 nations attended the meeting and gave insights into HPV and cervical cancer epidemiology and management in their countries.

## CERVICAL CANCER AND HPV: THE ESSENTIAL EPIDEMIOLOGY

### Xavier Bosch

#### Global perspective

Globocan data suggest that around 470 000 cases of cervical cancer are diagnosed worldwide every year [1]. While this is a far lower incidence than for breast cancer (1 050 000 diagnoses per year), cervical cancer remains the second most common cancer for women. Colon/rectal cancer, lung cancer, and stomach cancer, which can affect both men and women, all have lower incidences at 446 000, 387 000, and 418 000, respectively.

The relative incidence for different cancers varies significantly between regions. In North America, cervical cancer is not among the leading seven cancers diagnosed in women, nor among the top seven cancers that result in death.

A very different scenario exists in Central/South America (where cervical cancer is the second most frequent cancer diagnosis and cause of death), Africa (most frequent cancer diagnosis and cause of death), and Asia (second most frequent cancer diagnosis and fourth most frequent cause of death).

It is estimated that the lifetime risk of any woman experiencing cervical cancer is 3–4%. Yet age adjusted rates (AARs) vary from as high as 42.7 in Eastern Africa and 38.2 in Southern Africa, to as low as 5.8 in Western Asia [1].

#### Human papillomavirus

HPV is a small DNA virus that demonstrates significant genetic variation, with more than 100 types identified and sequenced to date. Two oncogenes associated with the virus have been identified, and are known as E6 and E7, which have the ability to degrade the proteins of the cellular genes p53 and Rb, thus interfering with essential mechanisms of cell proliferation and repair. A different section of viral DNA has been shown to be immunogenic and appears to generate a protective response.

The pathogenicity of the viruses varies: some types (such as 6 and 11) are linked to genital warts, while others (such as 16 and 18) are frequently seen in women diagnosed with invasive cervical epithelial abnormalities or cancer. Infection does not inevitably lead to pathological changes; regression occurs in some cases.

Interestingly, HPV prevalence among the general population is significantly higher in North-Eastern Africa (34.0%) and Central/South America (23.5%) than in other regions. In Asia, incidence is higher in the East (13.8%) and South-central (11.9%) areas than in Japan-Taiwan (7.4%) and South-East Asia (4.9%).

#### HPV and cervical cancer

Worldwide, a review of the prevalence of the 11 most common HPV types in HPV-positive cervical cancer cases (*n* = 2855) indicates that although frequencies varied, HPV 16 followed by HPV 18 were most commonly detected in Africa, Central-South America, South Asia, and North America. HPVs 45, 31, and 33 were also seen globally [2].

HPVs 16 and 18 appear to be most frequent in cases of high-grade squamous intraepithelial lesions (HSIL) or invasive cases of squamous cell caroinoma (SCC), although the relative incidence of different types varies in different regions [3].

#### Epidemiology and management

HPV is now established as a causative agent for cervical cancer, with 95% of cervical cancer specimens testing positive for HPV DNA using GP5+/6+ primers PCR. Cofactors that increase the risk of cervical cancer include high parity, oral contraceptive use, smoking, and HIV infection. Diet, low socioeconomic status, and other genital infections also appear to influence risk.

The variation in distribution and age-prevalence for different types of HPV varies between countries. However, the distribution of HPV types in cervical cancer shows universal dominance of HPVs 16 and 18, with some variability thereafter [4]. The 15 types that explain 95+% of cervical cancer do not show significant variability.

## HPV: VIROLOGY AND EPIDEMIOLOGY

### Ian Frazer

#### Human papillomavirus (HPV)

Papilloma viruses are double-stranded DNA viruses that replicate only in the skin cells of their host species. They do not grow in cell culture.

There are many different immunologically distinct types of HPVs, which have been linked to specific conditions—genital warts (HPVs 6, 11); genital cancer (HPVs 16, 18); epidermodysplasia verruciformis EV (HPVs 5, 8); and cutaneous warts (HPVs 1, 2).

HPV infects stem cells in the skin. Because the virus does not kill the host cell, it must wait for cell shedding (desquamation) to escape. The viruses do not generate an inflammatory response.

In cancer-causing HPVs, recognition by the immune system relies on growth-regulating segments known as E6 and E7. These proteins extend cell proliferation and retard cell differentiation, but do not inevitably result in malignancy. Cancerous transformation occurs only in vivo. On a cellular level, it is not known why some HPV types are more likely to induce cancer than others.

#### Cervical cancer: the HPV connection

HPV infection is common and is usually acquired soon after sexual activity commences. Most HPV infections regress without treatment. Less than one in 50 women infected with HPV 16 will show residual infection after 5 years. Progression of infection to precancer is slow and uncommon [5].

Yet every year, around 250 000 women die as a result of cervical cancer, and HPV is almost always present (99.8%). The most common type of HPV seen in cervical cancer is HPV 16 (∼ 60%).

#### Preventing cervical cancer

Currently, the only method of preventing cervical cancer is to identify precancerous lesions early and treat them. However, vaccines may prevent infection with high-risk HPV viruses, breaking the cycle far earlier.

Although the response is slow, HPV generates a type-specific immune reaction [6]. While this virus is not highly immunogenic, regression of HPV is dependent on this immune response—it occurs less frequently in immunocompromised patients such as renal transplant recipients. Details of this immune response are not completely understood, but humoral, cellular, and innate immunity may all play a role.

#### HPV serology and vaccination responses

The WHO recognised that without a standard reference serum to enable laboratories to standardise the calibration of HPV antibody testing, monitoring of vaccination programmes would be unreliable.

Test sera (particularly HPV 16) were distributed to 10 interested laboratories. Although all laboratories ranked the sera in the same order, there was significant variation in cut-offs for seropositivity. The study demonstrated that there was no universal ability to rank weakly reactive sera and to avoid cross reactivity [7].

Sufficient positive serum is being gathered to make a reference serum, which may be available by the end of 2006.

## HPV DNA TESTING: ASSAYS AND STANDARDS

### Suzanne Garland

#### Improving diagnostic testing for HPV

Laboratory procedures with known, consistent specificity and sensitivity for detecting and typing HPV are needed to conduct effective epidemiological surveys and vaccine evaluation. The standardisation of HPV assays is still evolving and requires external quality assessment.

HPV DNA testing may be used clinically for

- screening, either alone or as an adjunct to cytology,
- triage of patients with uncertain Pap results,
- monitoring patients post-treatment.

#### HPV detection

The presence of HPV can be identified by

- cytology (Pap smears),
- histology,
- electron microscopy,
- immunohistochemistry (identification of group-specific antigen),
- molecular tests (including in-situ hybridisation, dot blot techniques and others requiring amplification of viral DNA),
- serology (detection of capsid proteins or VLPs).

Amplification of viral DNA may be through target amplification (PCR) or signal amplification (Hybrid Capture II).

The Hybrid Capture II Assay (HC II) has been approved by the FDA and is available in microtitre format. An exfoliated cervical sample is assayed against different probe groups. This technique involves five specific stages and results are not type-specific, although low- and high-risk probe mixes are available.

PCR is highly sensitive, permits both detection and genotyping, and allows testing of different sample types including archival samples with poorer quality DNA. However, inhibitors in clinical specimens can lead to false-negative results, and contamination may result in false-positives. Most assays target the L1 region of viral DNA, but primers and detection systems vary.

ELISA-based microtitre plate format, reverse line blot/ strip assays, and microarray DNA chip assays can be used for PCR-based genotype-specific detection.

Roche has developed AMPLICOR HPV which uses a microwell plate-based ELISA technique to amplify and detect 13 high-risk HPV types.

In a preliminary study at the Royal Women's Hospital in Melbourne, Australia, HC II and AMPLICOR were compared by testing a range of samples; outcomes were compared to histology/cytology results. Both assays were positive for the 1 case of cancer, and of 38 samples with histologically confirmed HSIL lesions, Amplicor demonstrated an 89% sensitivity compared with 79% for HC II. The tests were less sensitive in predicting LSIL lesions: HC II and Amplicor detected 50% and 59%, respectively. The two tests had similar numbers of positives (∼ 9%) for those with normal histology, although these were a high-risk population, having previously had an abnormal Pap.

Line blot or line-probe assays can be used to recognise different genotypes of HPV. These PCR techniques are detailed and intensive.

#### Expanding use

HPV DNA testing is an objective test that will play an increasing role in the management of women with minor cytological abnormalities, as the results are more sensitive and reproducible than Pap smears in determining a woman's risk of having underlying HSIL, the precursor lesion for cervical cancer [8]. In combination with Pap cytology, the negative predictive value approaches 100%, resulting in fewer unnecessary colposcopies.

However, communication, education, and counselling will be required for women testing positive to HPV DNA. There is a high prevalence of HPV in clinically normal young women, and most infections are transient. Repeat testing is the sole means of determining HPV persistence.

#### Moving forward in a standardised way

International standard reagents provide a helpful tool for high-quality HPV amplification, detection, and genotyping. The WHO has a panel working towards standards that will enable laboratories to measure their results in relation to other groups.

Knowing specificity and sensitivity of particular HPV genotyping methods is crucial for epidemiological surveys, vaccine evaluation, and ongoing monitoring of vaccine performance following deployment. Complete protection against persistent HPV infection in vaccinated women has been demonstrated by two different HPV preventative vaccines in independent studies [9, 10].

HPV detection or even persistence is not an appropriate endpoint for preventative vaccine trials, as most infections are transient. Therefore, cervical intraepithelial neoplasia (CIN) of moderate or high grade are being used as the primary endpoint for vaccine trials. Once this surrogate endpoint has been proven, virological or immunological correlates of protection may be considered for future evaluation and product development.

## HPV TESTING IN CLINICAL PRACTICE

### Henry Kitchener

#### Potential roles for HPV testing in cervical screening

The most important clinical value of HPV testing in the lower genital tract is to distinguish women at very low risk of malignancy from those at some risk.

In cervical screening, the principal roles that HPV testing may play are

- in primary screening,
- in following up the treatment of cervical abnormalities,
- triage of mild abnormalities.

#### Study findings

The ASCUC-LSIL triage study (ALTS) investigated the sensitivity of HPV testing using HC II combined with repeat cytology in detecting CIN3+ lesions (Table 1) [11].

Further results from the ALTS study, in which 897 cases of low-grade squamous intraepithelial lesion (LSIL) and 1193 cases of HPV DNA-positive ASCUS were followed for 2 years, suggested that LSIL lesions and HPV-positive ASCUS are clinically equivalent [12]. Initial colposcopic detection of obviously prevalent CIN2+ reduces risk. However, for the remaining women who have CIN ≤ 1 on colposcopy and directed biopsy, the risk for subsequent CIN grade 2 or 3 is approximately 12% over 2 years.

The HPV in addition to routine testing (HART) study looked at the management of women who tested positive for high-risk types of HPV, but had negative or borderline screening abnormalities [13]. HPV testing was more sensitive than borderline-or-worse cytology (97.1% versus 76.6%, *P* = .002), but less specific (93.3% versus 95.8%, *P* < .0001) for detecting CIN2+. Surveillance at 12 months was as effective for monitoring progression as immediate colposcopy.

#### From study results to clinical practice

A meta-analysis to assess the accuracy of HPV DNA testing as an alternative to repeat cytology in women who had equivocal results on a previous Pap smear suggests that HC II assay has higher sensitivity and similar specificity than the repeat Pap smear (ASCUS as threshold) for CIN2+ among women in this patient group [14].

HPV testing has a high negative predictive value and although the positive predictive value is lower, it offers considerable benefits in triaging patients. It can also assist decision making on behalf of women with mild cytological abnormalities.

## BASELINE RESULTS OF HPV DNA TESTING IN EUROPEAN SCREENING STUDIES [15]

### Jack Cuzick, Christine Clavel, Ulli Petry, Peter Sasieni, Chris Meijer, Philippe Birembaut, Anne Szarewski, Achim Schneider, Shalini Kulasingam, Sam Ratnam, and Thomas Iftner

#### Objectives for meta-analysis

The objectives for reviewing HPV screening trials were to

- determine the age-specific HPV prevalence in different European areas,
- evaluate the sensitivity and specificity of HPV testing in women attending routine screening,
- compare the sensitivity and specificity of HPV testing with that of routine cytology.

#### Studies

**Figure 1:**
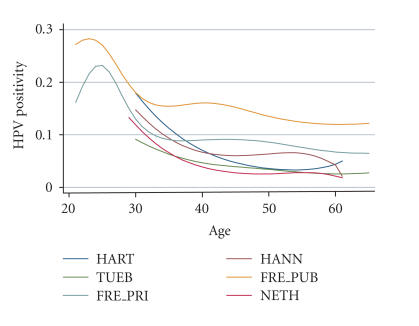
Age and HPV positivity.

Studies included were from the UK (HART [13], Hammersmith [16]); France (Reims [17]); Germany (Hannover and Tuebingen [18], Jena [19]); and the Netherlands (Amsterdam [20]) (Figure 1).

Comparisons were also made with North American screening studies reported by Ratnam et al [21] and Kulasingam et al [22].

#### Testing for HPV

Across the European studies, the HC II test for HPV had a sensitivity of 96% and specificity of 92%, whereas cytology based on Pap smears had a sensitivity of 63% and specificity of 96%.

Although the performance of HPV testing is similar in different areas of Europe, the sensitivity of cytology is highly variable between countries.

#### HPV as a sole primary screening test

There are a number of potential advantages in using HPV testing (HC II) as the sole primary screening test for cervical cancer. Due to its automated, highly sensitive, and objective nature, results are less variable and quality control is simpler. Cytology could thus be reserved for 6–10% of women; screening would be undertaken in more detail by a smaller number of more focused cyto-screeners. Triage of HPV-negative ASCUS/LSIL lesions would be eliminated.

Screening intervals could be longer with potential cost savings and greater convenience.

## TREATMENT OF PREINVASIVE CERVICAL LESIONS: IS THERE ANY VALUE IN TESTING FOR HPV DNA POST-TREATMENT?

### Jeffrey Tan

#### Treating preinvasive cervical lesions

A 2001 Cochrane report suggests there is no overwhelmingly superior surgical technique for eradicating CIN [23].

Regardless of treatment approach, the risk of persistent disease is greater when the lesion is large, or the patient is > 30 years of age or has been treated previously and/or is a carrier of HPV types 16 or 18 [24].

#### After treatment for CIN

Between 1998 and mid-2003, 3647 women were treated at Melbourne's Royal Women's Hospital (RWH) for CIN.

Among those who returned 3–6 months postsurgery, 21% returned an abnormal Pap smear/histology. By 9–14 months, persistent low-grade abnormality was seen in 9.4% and high-grade abnormality in 0.7% of women.

Residual disease following large loop excision of the transformation zone (LLETZ) surgery may be missed in screening when cytology is used. In a 2002 review of 90 women, cytology had a positive predictive value of 81.3% and a negative predictive value of 55.4% [25].

Results from RWH comparing cytology and colposcopy for detecting SIL post-operatively suggest that cytology is more specific than colposcopy (82.6% versus 24.4%), but far less sensitive (46.0% versus 90.5%).

As cytology and colposcopy both present difficulties in sensitivity and specificity, HPV testing may provide an alternative approach. A review of 11 studies published from 1992 to 2002 demonstrated that among 900 women treated for CIN, 204 had residual or recurrent cellular abnormalities and incidence of HPV was 83%; among 696 women with apparently successful outcomes, the incidence of HPV DNA was only 15% [26].

A meta-analysis of 11 studies suggests that the combination of HPV testing with cytology is promising for post-treatment evaluation [27].

#### The value of HPV DNA testing post-treatment

A study currently underway at RWH aims to determine whether HPV DNA (HPV HC II test) is a marker for recurrent disease following surgical management of CIN. Since 2001, over 1500 women have been recruited at the time of surgical treatment for CIN. Overall, 64% of the women had high-risk HPV detected at surgery. For women with HSIL confirmed histologically at surgery, 79% had high-risk HPV DNA detected on the hybrid capture test.

These women are reviewed regularly and at each visit Pap and HC II tests are undertaken and colposcopy performed.

The overall positive rate for high-risk HPV DNA fell from 31% at 3–9 months to 20% by 21–25 months post-treatment. If Pap cytology and HC II were negative 12 months after treatment, only 0.6% of women had HSIL.

This study is still ongoing, although these preliminary results suggest that the negative predictive value of high-risk HPV DNA holds promise as a marker for the adequacy of HSIL treatment when performed ≥ 12 months post-surgery.

Revised guidelines for post-treatment assessment of cervical lesions will be introduced in Australia in July 2006. Colposcopy and cytology should be undertaken 4–6 months after treatment for HSIL. Cervical cytology and HPV typing will be used at 12 months, then annually until negative results for both tests are obtained on 2 consecutive occasions. From that time on, Pap smears will then be at the recommended screening interval of 2 yearly.

## SINGAPORE: CANCER SCREENING AND EPIDEMIOLOGY

### EH Tay

#### Assessing benefits, risks, and costs of cancer screening

In Singapore, cancer causes more deaths than heart disease [28]. Cervical cancer is the fourth most prevalent cancer in Singapore, and based on 1993–1998 data, has an ASR of 14.3/100 000. Breast, lung, colorectal cancers, and, in recent years, ovarian cancers, have a higher incidence [29]. Breast, colorectal, and ovarian cancer rates are increasing; lung and cervical cancer are decreasing, a trend believed to be linked to behavioural factors [29].

Current cervical screening recommendations in Singapore recommend Pap smears every 3 years for women from time of first intercourse or from 25 years of age until 65 years. A cervical screening programme for Singapore was first proposed in 1992; however, breast screening was given priority. In 1997, a plan for a pilot study was submitted and accepted by the Health Promotion Board. Following 5 years of development, the pilot programme is currently underway. Roll-out nationally had begun in 2004.

Short-term challenges for the project are to

- ensure coverage across the population, through education of doctors and women
- prepare laboratories and standardise reporting—a local systematic reporting system has been adopted,
- effectively manage and treat women with abnormal smears,
- address the issue of false-negative Pap smear results.

Past failures of cervical screening [30] attributable to lack of adequate quality control, rather than to technological limitations of the Pap test, has shifted the focus from new technology (such as automation) [31] toward quality assurance [32]. Retrospective review has highlighted areas for laboratory education and quality improvement efforts, and strong liability concerns have prompted the introduction of governmental regulation of laboratories, including obligatory accreditation.

#### Prophylactic vaccination

HPV vaccination has the potential to become the second prophylactic vaccine capable of reducing the incidence of cancer, following the success of hepatitis B vaccine in reducing hepatocellular carcinoma in some areas of the world [33].

Two vaccines for HPV are currently in late-stage development. However, decisions on vaccine types, efficacy, and usage will await the outcomes of trials currently underway, such as the FUTURE II study of a quadrivalent vaccine in young women in the USA and Brazil, and the testing of a bivalent vaccine by the GlaxoSmithKline HPV Vaccine Study Group.

As a primary prevention, vaccination offers the advantage of being able to be administered without specific training. In the long-term, vaccination against oncogenic HPV should reduce the incidence of persistent infection and subsequently of cervical cancer mortality. As part of a screening programme, the role and cost-effectiveness of prophylactic vaccines will be influenced by age at the time of vaccination, level and duration of immunoprotection, epidemiology of local HPV types, and cost of the vaccine [34]. In Asia, while squamous cell cancer is most frequently associated with HPVs 16 and 18, which are predominant in most countries of the world, there is some evidence that HPV 52 and 58 are more prevalent [35].

Considerations for planners include

- uncertainty about the role and response of antigenic HPV subtypes in cervical cancer causation,
- the impact vaccination will have on frequencies of nontargeted HPV types,
- the need to vaccinate men as well as women.

The impact of HPV vaccination on cervical cancer incidence would not be immediate. If vaccination of young girls begins in 2010, it would be almost 20 years before the decline of CIN and cancer incidence may become apparent, in 2030.

Singapore plans to implement universal Pap smearing from 2006; in light of the current breakthroughs, a strange irony has emerged. While the implementation of a national cervical cancer screening programme brings the exciting prospect of controlling a largely preventable cancer, the country must still face the costly process of preventing misdiagnosis due to the less-than-perfect sensitivity and specificity of the screening Pap test. The use of HPV testing and vaccination holds significant promise, but will not eliminate the need for Singapore to address quality control in Pap smear collection, cytology, and reporting [36].

#### Therapeutic vaccination

Preliminary results suggest that vaccines may also have therapeutic benefits in women showing precancerous CIN lesions [37, 38]. It appears that vaccines may stimulate the immune system and cause the regression of precancerous CIN 1, CIN 2, and CIN 3 lesions when given locally.

## ORGANISATIONAL ASPECTS OF SUCCESSFUL SCREENING PROGRAMMES IN DEVELOPED COUNTRIES: THE ENGLISH MODEL

### Henry Kitchener

#### Assessing benefits, risks, and costs of cancer screening

In planning a cancer screening programme, each country will develop guidelines based on the best available estimates of costs, benefits, and risks. Guidelines can be reviewed in light of emerging evidence as well as new diagnostic and treatment options.

Evaluation of a programme's coverage should consider the age of eligible participants, the frequency of testing, and community awareness of, and access to, the programme. All will impact on cost and effectiveness.

Recommendations on the age of initiation and frequency of cervical screening vary between European countries. Van Ballegooijen et al compared the relative cost-effectiveness of programmes in European countries based on recommended screening age ranges and intervals and coverage (Table 2) [39].

Sasieni et al used UK data to compare the incidence of cervical cancer in different age groups of women against the number of years since their last negative screening result [40]. His findings revealed that less frequent screening may confer greater advantages in older women (> 40 years) compared with younger women. He found that 5-yearly screening offers considerable protection (83%) against cancer at ages 55–69 years; annual screening provides only modest additional protection (87%). On this basis the cervical screening programme now offers 3-yearly and 5-yearly screening for women aged 25–50 and 50–64 years, respectively.

The proportion of women in the community who are screened at the recommended frequency will also influence any evaluation. In 2004, around four in every five British women aged 25–64 years had been screened for cervical cancer within the previous 5 years.

Primary care practices carry the prime responsibility for the continuity of testing. A review of primary care organisations in 2004 showed that coverage of eligible women in different practices varied from 70% to 90%.

Training and evaluation programmes in cytology and colposcopy have been established. The majority of British laboratories reviewed in 2003–2004 achieved 65–85% positive predictive values in their reviews of cervical smears (the proportion of high-grade cytology associated with underlying high grade CIN).

National computerised colposcopy data is not routinely collected; for example in 2003–2004, more than 70% of women with abnormal screening results saw a specialist for colposcopy within 8 weeks. This was followed by a diagnostic biopsy in 40% of cases, and 19% had lesions excised on that first visit. In 37% of referred patients, colposcopy appeared normal and no procedure was required.

New options such as HPV DNA testing, the availability of liquid-based cytology and a growing awareness of the relatively small benefits gained through frequent testing for women < 40 years of age, are likely to influence the evolution of the British screening programme.

## CERVICAL CANCER IN THE PHILIPPINES

### Genera A. Manuel-Limson

#### Epidemiology and screening

In the Philippines, cervical cancer is the second most common cancer among women, behind breast cancer [41]. The age standardised rate (ASR) is estimated to be 22.5/100 000. Incidence rises sharply in women > 35 years of age. More than 7000 new cases and almost 4000 deaths are seen each year.

There is a close link between neoplastic cervical changes and HPV persistence. Of 356 cases of SCC or ADC, 93.5% tested positive for HPV, compared with only 9.1% among a control group [42]. HPV types 16 and 18 were most frequently associated with cervical cancer.

As two in every three cases of cervical cancer are detected in late stages of the disease, the median survival rate after diagnosis is only 76 months; 5-year survival is 51.7%.

#### Cervical screening in the Philippines

The Philippine health infrastructure is not sufficiently developed to support a well-structured, cytology-based screening programme. Pap smears are available through family planning clinics and relevant societies; however, screening is not coordinated or appropriately targeted. Alternative strategies for lowering the cervical cancer disease burden must be considered.

#### Cervical cancer screening study group

The Department of Health and the Medical Faculty at the University of the Philippines reviewed screening options to identify an approach that is feasible, cost-effective, and replicable, to reduce the need for extensive cytology services and radical forms of treatment.

The study focused on

- knowledge, attitude, practice, and behavioural modification,
- validity and reliability standards for the screening test,
- cost-effectiveness,
- health policy implications.

Four approaches to screening were compared:

- unaided acetic acid visualisation (AA),
- magnified acetic acid visualisation (MAA),
- Pap smear using spatula (S),
- Pap smear using cervical brush (CB).

Women 25–65 years of age were recruited for the study and of those screened and interviewed, 13 105 underwent colposcopy and were included in the data analysis.

#### Results

The results demonstrated the potential for acetic acid visualisation in a screening process (Table 3).

As a result of this trial, the study group recommended that

- the acetic acid aided visual method be used as the initial screen for cervical epithelial abnormalities at health centres where Pap smear is not available,
- all women showing abnormalities be referred for colposcopy, and biopsy, if necessary.

Screening coverage for Filipino women is still low due to inadequate healthcare personnel and a shortage of facilities. There has been no sustained public health campaign to promote the benefits of regular screening. With many other pressing public health priorities in the Philippines, funding for cervical screening is limited.

## INDIA: EPIDEMIOLOGY AND SCREENING OF CERVICAL CANCER

### Neerja Bhatla

#### Epidemiology of cervical cancer in India

Cervical cancer is the most common neoplasm in Indian women, with 126 000 new cases and 70 000 deaths each year. Incidence is higher than in Eastern Asia [1].

Across India, AARs vary greatly from ∼ 55/100 000 in Ambillikai to < 18 in Mumbai and Trivandrum [43]. Across India, cervical cancer is the commonest cancer reported from all cancer registries except those in Mumbai and Delhi, where breast cancer is the commonest.

#### HPV and cervical cancer

Persistent infection with HPV has been linked to almost all cases of cervical cancer and 73–85% of CIN [44, 45].

In India, most studies previously focused on the prevalence of HPVs 16 and 18 in the general population, which was reported to be 7.5–9.6%—lower than global estimates—but recent studies by IARC that looked at all high-risk types have reported prevalence rates of 14–19%. Among Indian women with CIN, 73% tested positive for HPV [46]. Types 16 or 18 were present in 73–82% of cervical cancer. However, a systematic study for all HPV types found 99.5% of tumours to be positive for HPV [47]. In another study from the All India Institute of Medical Sciences (AIIMS), 98.1% of tumours were positive for HPV. The most common types in these two studies were HPVs 16, 18, 33, and 45.

**Figure 2:**
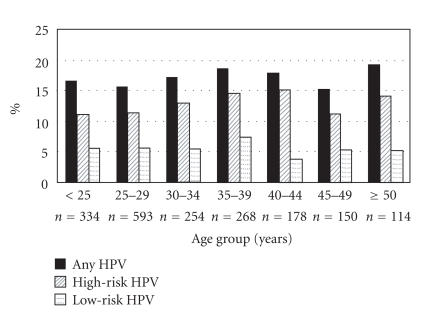
Age distribution of oncogenic HPV types in an Indian population [48].

The highest prevalence of HPV infection was previously reported to be between 21–24 years of age, but more recently, a systematic IARC study has shown that there is no distinct age-related peak for HPV prevalence (Figure 2) [48].

In a 2005 survey of HPV genotypes at the AIIMS in New Delhi, HPV 16 was by far the most prevalent type, detected in more than 35%, while HPV 18 prevalence was ∼3%.

#### Cervical cancer screening in India

The National Cancer Control Program has operated since 1972. Although cervical cancer is a stated priority, there has been no coordinated cytology screening.

The current recommendation of the Indian Council of Medical Research is that all women should have a single Pap smear at approximately 35 years of age. In reality, screening is opportunistic, frequently in research settings.

Developing countries must rely on less sophisticated resources, such as visual inspection with acetic acid (VIA), visual inspection with acetic acid magnification (VIAM), or visual inspection with Lugol's iodine (VILI).

A series of trials has evaluated the efficacy of these approaches in detecting high-grade disease. Results have been very variable, with VIA having a sensitivity of 55 [49]–88% [50, 51] and specificity of 63 [51]–94% [52]. Comparisons between combinations of VIA, VIAM, and VILI have been reported by Basu et al [49], Sankaranarayanan et al [53], the IARC [54], and Shastri et al [55] with variable results.

In screening for cervical cancer, VIA offers the following advantages over alternatives:

- simple, easy-to-learn approach,
- low startup and ongoing costs,
- less reliance on infrastructure or medical specialists to perform procedure,
- immediacy of results,
- potential for integration into primary health care services.

These need to be weighed against the disadvantages:

- moderate specificity—resulting in higher referral and potential over-treatment,
- dependence on the person doing the evaluation—need for standard training methods and quality assurance,
- lower accuracy in postmenopausal women.

#### Is there a role for HPV testing?

In detecting high-grade cervical lesions, the role of HPV testing remains unclear. The test could be undertaken using samples collected by the patient. In a recent trial by AIIMS, 93.5% of participants provided a satisfactory self-sample, and concordance between physician-collected and self-samples was high (93.8%).

HPV testing can be automated and provides a standardised, objective result. In combination, it can improve the sensitivity of Pap smears and VIA.

However, major problems for the widespread adoption of HPV testing in India are its cost, the need for sophisticated laboratory infrastructure, and repeat visits.

#### The future of screening in India

Newer technologies may increase the specificity of testing without loss of sensitivity, but are currently too costly for generalised adoption.

Point-of-care diagnostic tests such as VIA and the “rapid” HPV test will benefit populations with poor compliance.

## CHINA: HPV INFECTION AND CERVICAL CANCER SCREENING STUDIES

### You-lin Qiao

#### Epidemiology of cervical cancer in China

Mortality data from the 1970s to the 1990s suggest that China suffers relatively high cervical cancer mortality, particularly in the rural mid-west. Wudu in Gansu province and Yangcheng in Shanxi had age-adjusted mortality rates of > 40/100 000 in 1990–1992.

Better diagnosis and treatment have reduced deaths from cervical cancer, but the improvement is not uniform and mortality rates are unchanged in some counties.

#### Status of cervical screening

There are no national screening programmes for cancer in China. In the case of cervical cancer, limitations include the nationwide shortage of cytologists.

In late 2003, a national cancer prevention and control strategy was finalised and endorsed by the Ministry of Health, following consensus meetings for early detection and treatment across nine cancer types.

Two demonstration centres for cervical cancer prevention and control have been established in Shenzhen (South-East China) for high resources settings and Xiangyuan (North-West China) for low resources settings [56]. A government recommendation that all women should have at least one screen between 35 and 65 years of age is still pending.

#### Cervical cancer research in China

The first Shanxi Province Cervical Cancer Screening Study (SPOCCS I) included 1997 women who underwent a cervical evaluation using HPV self-test, optical biopsy, liquid-based cytology (ThinPrep), VIA, direct testing for HPV, or colposcopy with biopsy [57].

A second study (SPOCCS II) included > 8000 women who submitted a self-sample for HPV at their village and subsequently visited the clinic for HPV Direct Test, LBC (AutoCyte), and VIA [58]. Those with abnormalities (*n* = 3252) underwent colposcopy and biopsy. Women with CIN 2+ lesions were treated; those with CIN 1 lesions will be followed up in 12 months.

In both studies, women with cervical cancer or CIN 2+ lesions on biopsy were likely to be infected with HPV (> 95%). Even for women with CIN 1 lesions, the attributable risk of HPV is as high as 95%. The incidence of HPV among women with normal biopsies was < 15%.

Persistent HPV infection was higher among women aged 50–54 years than younger women in Shanxi, but there were marked differences in the age-related curves for women in rural areas versus cities.

#### Risk cofactors

Extensive lifestyle data were collected covering sexual history, child bearing, occupation, health, bathing practices, age at menarche and menopause, education, and income.

Lifestyle had a significant effect on HPV and cervical cancer prevalence in Shanxi. Risk factors that appeared to contribute to the likelihood of cervical cancer were subject promiscuity (OR = 1.42); husband's promiscuity (1.42); bathing at a public house (1.23); postmenopause (1.22); and current smoking (1.17). Surprisingly, education appeared to slightly raise the likelihood of HPV.

When self-testing was compared with direct testing for HPV, specificity for CIN 2+ was identical (85.9%), while sensitivity was higher on direct testing (97.6%) than for self-testing (83.5%).

#### Evaluation of screening tests

In both trials, each screening test was compared with pathology to determine accuracy in detecting moderate-high-grade lesions (Table 4).

#### Conclusions

With high sensitivity (> 96%) and moderate specificity (86%), the HPV direct test could be used as a primary screening test for cervical cancer risk. Self-sampling is simple and less expensive; however, improvements in instructional leaflets will be needed.

Combined HPV and VIA offers potential for screening in the mid-west regions of China, but for consistent results, VIA training will be essential.

## HONG KONG: CERVICAL CANCER SCREENING

### Annie NY Cheung

#### Women and cervical cancer

Hong Kong has a population of 6.8 million. Life expectancy for women at birth was estimated to be 84.3 years in 2003.

**Figure 3:**
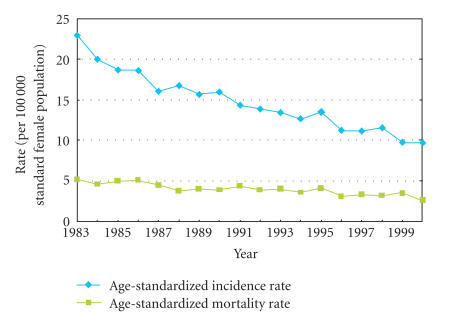
Cancer of the cervix—age-standardised incidence and mortality.

During the period 1988–1992, Hong Kong's ASR for cervical cancer was ∼17; this is lower than that of the Thai, Korean, Filipino, and Singaporean-Chinese populations, but higher than in Japan (Figure 3) [59].

#### History of cervical screening

Until 2004, there was no centrally organised, systematic cervical screening programme (CSP) in Hong Kong. Screening was generally opportunistic or included as part of a general checkup. Screening practices varied between different healthcare providers and there was little collaboration between public and private sector healthcare.

Approximately 45% of women, primarily those who were educated and health-conscious, were being screened. This somewhat random coverage was not equitable or efficient, and was unlikely to be cost-effective.

Despite this, the ASR for cervical cancer declined steadily between 1983 and 2000, the most significant fall being among women 50–65 years of age. About 50% of cases are diagnosed at stage 1.

#### A centralised cervical screening programme for Hong Kong

First approved in 2001, the Hong Kong CSP was launched in March 2004. The goal is to reduce the incidence of, and mortality from, cervical cancer by facilitating regular screening for all patients at risk.

Stated objectives are to

- raise public awareness,
- improve population coverage,
- promote more equitable and efficient screening,
- build quality assurance into the services through professional education and clear guidelines,
- support private sector activity.

Following statistical modelling, the target population was set to include all women 25–64 years of age who have had sex. After two clear annual checks, screening will be scheduled every 3 years, and discontinued at 65 years if the previous Pap smears are normal.

Using triennial screens, it was estimated that if coverage raised to 80% of the population, there would be ∼75% decrease in the number of cases of cervical cancer.

It is hoped that the CSP will increase coverage from 43% to 60% within 3 years, and to 80–85% in the long term.

During the period March 2004–June 2005, almost 150 000 women were enrolled in the CSP. Based on age quintiles, enrolment has been highest among women 40–44 years of age. There are large variations between districts.

#### The communication network

The CSP incorporates a centralised information system with a website (available at http://www.csis.gov.hk) accommodating the input of data online.

The central registry will include screening results, followup investigations, and demographic data, and will generate reminder letters to women due for screening. Practitioners will be emailed details of patients recalled, and alerted to abnormal smears. The system will also provide data for ongoing evaluation and monitoring.

Public education regarding cervical cancer has not been a priority in Hong Kong; therefore, communication and promotion are priorities. Attention was drawn to the disease when Anita Mui, a popular singer and movie star, died from cervical cancer in December 2003. She was 40 years of age. Her death coincided with the launch of the CSP and may have aided receptivity.

#### Quality assurance

All registered doctors and trained nurses may collect cervical smears. Accreditation for participation is coordinated by the Society for Colposcopy and Cervical Pathology. Health professionals whose smears do not meet CSP minimum standards will be invited to attend refresher training.

Healthcare professionals who supply smears to the CSP are provided with a training kit, which covers technical skills and communication approaches.

A survey of women will be undertaken to gauge levels of satisfaction with the CSP and the Pap smear.

The Hong Kong College of Obstetricians and Gynaecologists (HKCOG) has developed guidelines for the taking of Pap smears and management of abnormal smears [60]. Other reference guidelines for pathology [61] and cytology [62] have also been prepared.

Cervical cytology laboratories operating under the CSP must be approved by international bodies and the Hong Kong laboratory accreditation scheme.

#### Testing and triage

The CSP uses Pap smear cytology as the primary screen.

Among smears undertaken to date, 5.6% have shown epithelial cell abnormalities. Of the abnormal smears, 60–80% were recorded as ASCUS. ASCUS patients were either referred for colposcopy or followed up with a further screen. However, only 2–10% of ASCUS patients have serious disease [63].

HPV testing is currently under consideration as a means of triaging patients with ASCUS and low-grade lesions.

A trial was undertaken where ASCUS smears were repeated (*n* = 5579); on second review, 9.8% and 1.7% showed LSIL and HSIL lesions, respectively [64]. Patients with a primary ASCUS smear are at greater risk of developing SIL and should be followed up.

A study of patients with ASCUS smears checked HPV status using HC II testing. To date, 2309 samples have been screened. Almost half (47.9%) were positive for high-risk HPV strains. HPV-positive women with ASCUS were more likely to have HSIL (*P* = .001) and LSIL (*P* < .0001) detected in their next cervical cytology samples.

Despite its benefits, HPV typing remains expensive. In addition, anxiety is generated when a woman is told that she has been exposed to the virus. To ensure that information is communicated effectively to patients, it is important that incorrect conclusions are avoided. For example, having been told that cervical cancer is caused by HPV, an STD, patients should not conclude that cervical cancer is an STD. Similarly, although sexual promiscuity increases the risk of HPV and cervical cancer, an HPV-positive test does not imply that a woman has been promiscuous. These communication challenges mean that HPV may not be an acceptable primary screening test.

## SCREENING TECHNOLOGIES TO ADVANCE RAPID TESTING (START)

### John Sellors

#### START objectives

This project aims to detect precancerous cervical lesions using newly developed rapid biochemical tests that are affordable, accurate, simple to use, and appropriate for low-resource settings.

PATH is currently working on two promising candidates:

- batch test (46 samples) for use in a small clinic or mobile unit (results in ∼2 hours) in collaboration with Digene Corporation (USA),
- rapid strip test for a near-patient setting (results in less than 20 minutes) in collaboration with Arbor Vita Corporation (USA).

Following three years of research and development on the new tests, the goal is to have a prototype for testing (verification, validation, field tests) in 2006 and 2007. PATH has negotiated an agreement with each private-sector partner to supply the tests at a preferential price to the public sector in developing countries for a period of ten years.

#### The tests: current progress

The rapid batch test developed by Digene Corporation will use an instant photo signal output. Images of samples will be compared visually on a film with positive and negative controls.

The rapid strip test promises to differentiate between transformation and infection by HPV. Arbor Vita Corporation technology detects a biomarker (E6 oncoprotein) which correlates with neoplastic transformation of cells and maintenance of cervical cancer. The ELISA prototype is now being adapted to an immunochromatographic strip format capable of detecting common high-risk HPV types. Efforts are focused on improving sensitivity.

In addition to using a cervical sample obtained by a health care provider, vaginal sampling by a woman herself or a provider is being investigated for both assays.

#### Collaborative arrangements

Clinical work will be undertaken in China (Cancer Institute Chinese Academy of Medical Sciences, Beijing, will coordinate evaluation across several provinces) and India (Tata Memorial Hospital, Mumbai, will oversee testing in the state of Maharashtra).

Participation in the project offers benefits for the collaborating countries:

- approximately 22 000 rural women will be screened for cervical cancer and treated, if necessary;
- the project will provide job opportunities in outlying districts;
- biomedical workers will have opportunities for intellectual exchange;
- improved tests should provide a more affordable, accessible, and acceptable screening option. Better population coverage would lower disease incidence and mortality.

#### Moving forward

Throughout development collaborative input has been key, with contributions from users (both the “tested” and testers), private-sector partners, policy makers, and economists.

For more information on the START project go to http://www.path.org/projects/start_project.php.

## COSTS OF HPV DNA TESTING IN CERVICAL SCREENING

### Jack Cuzick

#### Introduction

HPV DNA testing has an estimated sensitivity of 96% and specificity of 92%, making it considerably more sensitive and only marginally less specific than cytology. There is sufficient evidence based on surrogate markers that the efficacy of HPV testing, using a validated system, as the primary screening modality can be expected to be at least as good as that of conventional cytology [68].

#### HPV testing: potential role in primary screening

HPV testing offers a number of advantages when used in combination with cytology in primary screening:

- higher sensitivity,
- longer screening interval,
- fewer inadequate samples.

If HPV testing were the sole primary screening test, cytology could be used to triage patients who test positive. Self-sampling may improve coverage.

#### Potential cost impact of replacing cytology with HPV testing

Cost reductions would result from longer screening intervals, fewer inadequate smears, and avoidance of borderline smears in women not infected with HPV. However, lab costs would be higher and surveillance rates would rise.

The cost impact of adding HPV testing to the British cervical screening programme, and increasing the screening interval from 3 to 5 years, was estimated in 1998 [69]. The result suggested a fall in costs of around £30 million, or almost 25%.

## EMERGING PREVENTION STRATEGIES: PROMISES OF THE QUADRIVALENT HPVS 6, 11, 16, 18 VACCINE (GARDASIL)

### Richard M. Haupt

#### Rationale for quadrivalent vaccine development

A vaccine protecting against HPV types 6, 11, 16, and 18 is expected to substantially reduce the burden of HPV-related diseases.

Merck's quadrivalent HPV L1 virus-like particle (VLP) vaccine, GARDASIL, has been well tolerated, immunogenic, and effective against HPV infection in early studies. Phase III studies are underway to definitively evaluate the clinical and public health impact of GARDASIL in adolescent and adult men and women (Table 5).

#### Vaccine profile

The quadrivalent vaccine comprises VLPs produced in a recombinant yeast [70]. The vaccine is adsorbed on the Merck proprietary aluminium adjuvant, which strengthens its immunogenicity.

Injection volume is 0.5 mL. Boosters are given at 2 and 6 months.

#### Clinical programme

The three-phase development programme is moving towards completion.

- Phases I and IIa: preliminary assessment of immunogenicity and tolerability of different doses of monovalent HPV L1 VLP vaccines.
- Phase IIb: immunogenicity and tolerability of different quadrivalent vaccine dose formulations.
- Phase III: demonstration of risk reduction for acquisition of HPV infection and development of genital warts and CIN 2/3 related to HPV types.

#### Results to date

*HPV 16 vaccine proof-of-principle study*

This early stage trial was double blind and placebo controlled [9]. Erolment involved 2391 US women aged 16–23 years, regardless of HPV status, who were followed for 4 years. Efficacy evaluation considered only women who were HPV 16-naïve at baseline.

The primary endpoint of the study was persistent HPV 16 infection (positive vaginal or cervical swabs on ≥ 2 consecutive visits), or HPV 16-related CIN (low-grade or high-grade precancer on a tissue specimen from an abnormal area on the cervix AND detection of HPV 16 virus in the same lesion), with an additional corollary endpoint of single HPV 16 detection at last visit on record.

After 4 years, among those vaccinated there were seven instances of HPV detection or CIN, compared with 111 cases among the placebo group. This data demonstrate an efficacy level of 94% (*P* = 10^−12^). Significantly, all seven cases in the vaccine group were of single HPV detection at last visit on record. The adverse event profiles were similar in the vaccine and placebo groups.

In tests undertaken at 7 months, all vaccinated women had significantly higher levels of specific serum antibodies than those on placebo, including those naturally infected [71].

*Dose-ranging and efficacy study*

More than 1100 women aged 16–23 years, from the USA, Brazil, and the EU, were enrolled in this double-blind, placebo-controlled study, and followed for 3 years [70].

Three formulations of quadrivalent HPV vaccine or placebo were given at enrolment, 2 months and 6 months.

Immunogenicity was reviewed against each viral type and women were monitored for persistent HPV in cervical samples, CIN+ lesions, and genital warts.

Antibody response proved similar with the three vaccine formulations, and all women given the vaccine had antibody titres greater than those seen following natural infection.

The lowest dose combination has become the standard formulation for GARDASIL.

Overall, four vaccinated women had persistent HPV infections (HPV 16, 18), compared with 36 women given a placebo vaccine. Efficacy was 90% (*P* < 10^−3^).

*Adolescent immunogenicity substudy*

If HPV vaccine is to be used prior to sexual debut, an adequate immune response must be demonstrated in adolescents.

A randomised, double-blind, multicentre study has been undertaken to compare immunogenicity, seroconversion, and safety in 10–15-year-old males and females, and 16–23-year-old females.

In all test groups, seroconversion levels at 7 months were higher than the results recorded in the earlier adult trial.

*Phase III*

Phase III studies will include ∼ 20 000 female patients at 150 sites across 33 ethnically diverse countries. Followup will be for 4 years from first dose.

The study in women began in 2001. A separate series of studies will be conducted to evaluate the vaccine's efficacy in men (heterosexual and homosexual). Men are a vector for HPV in women, and suffer from genital warts and anal cancer (AIN is increasing among gay men).

The studies are characterised by an inclusive centralised cervicovaginal evaluation programme. Women undergo Pap testing at 6–12 month intervals.

#### Looking ahead

Prophylactic vaccines are the most efficient means to reduce the clinical impact of infectious disease. If proven safe and effective, a quadrivalent vaccine targeting pathogenic HPV types will greatly reduce the burden of HPV-related diseases.

Preliminary studies for GARDASIL are promising, but the Phase III programme will provide a definitive assessment of the clinical utility of the vaccine.

## HPV 16/18 PROPHYLACTIC CERVICAL CANCER VACCINE: DEVELOPMENT UPDATE

### Hans Bock

#### Rationale for development

HPV types 16 and 18 are most frequently associated with cervical cancer, occurring in > 70% cases globally [72].

In some locations (the Philippines, Costa Rica, Bangkok), the odds ratio associating HPV and cervical cancer is > 10 times that of cigarette smoking and lung cancer [73].

The objective for vaccine development was to prevent persistent infection with HPV 16/18, and thus avoid abnormal cytological and neoplastic changes in the cervix.

#### GlaxoSmithKline biological HPV vaccine

GlaxoSmithKline has produced an HPV vaccine based on a recombinant L1 protein, which self-assembles into VLPs. These resemble intact viruses but are not infectious. The vaccine, currently in clinical trials, is the result of an early-stage collaboration between MedImmune and GSK.

Originally, the vaccine was produced using an aluminium adjuvant, but in Phase II trials a new adjuvant (AS04) generated faster, stronger, and longer-lasting antibody responses against both HPV types. Seroconversion remained at 100% for 24 months. The difference between the earlier aluminium adjuvant and AS04 was statistically significant.

#### Early studies

All formulations and dosage levels tested in early trials were well-tolerated, although local injection site reactions were common. Vaccination generated high levels of HPV 16/18-neutralising antibodies and CMI responses, particularly the formulations using the AS04 adjuvant.

The first efficacy trial, a double-blind, controlled, randomised trial, was conducted in the USA, Canada, and Brazil. More than 1100 women were enrolled; they were aged 15–25 years, claimed no more than six lifetime partners, and were seronegative for HPV 16/18 and tested negative for HR-HPV DNA in cervical scrapes.

The vaccine schedule included three doses (0, 1, and 6 months) and participants were monitored for 18 months.

The objectives were to

- evaluate efficacy against incident HPV 16 and/or 18 infection,
- evaluate efficacy against persistent HPV 16 and/or 18 infection and the development of HPV 16- and/or 18-associated cytologic and histologic lesions,
- determine vaccine safety, tolerability, and immunogenicity.

At 7 months, all vaccinated women showed ELISA responses that far exceeded those seen in naturally infected women. No instances of persistent infection were recorded in vaccinated women [74].

In the vaccine group, there was also statistically significant protection against HPV 31, 52, and 45. Types 31 and 52 are phylogenetically related to HPV 16, as HPV 45 is to HPV 18, yet this was the first evidence of cross-protection between HPV types. The GSK vaccine provides protection against high-risk HPV types in addition to HPV 16/18. Cross-protection increases vaccine coverage against cervical cancer [72].

#### Phase III efficacy studies

A large Phase III efficacy study will enrol 18 000 women, 15–25 years of age in 14 different countries across four global regions. It will be a double-blind, randomised, controlled trial over 4 years, with Independent Data Monitoring Committee (IDMC) oversight. Cervical samples for PCR and cytology will be taken every 6 months for 4 years. The objective is to gauge the vaccine's efficacy in preventing CIN2+, AIS, and invasive cervical cancer resulting from persistent infection with HPV 16/18.

The National Cancer Institute will undertake a separate study that will test ∼12 000 women through a single centre in Costa Rica. Data management will be overseen by an IDMC.

The programme also includes studies that will extend vaccination age coverage to 10–55 years. Key data is accumulating to support filing for launch in 2006.

Long-term studies to evaluate the vaccine's efficacy against cervical cancer are planned until 2015.

## IMMUNOTHERAPY FOR HPV: WHAT IS NEEDED AND WHY

### Ian Frazer

#### Preventing cervical cancer

The only approach to preventing the consequences of persistent HPV infection currently widely available involves treatment following early detection of CIN. This requires regular screening using Pap smears, visual inspection, and/or HPV testing, followed by destructive therapy to kill cancerous cells.

Prophylactic vaccines now in development use VLPs to prevent infection with specific HPV types.

It may be possible to use viral nonstructural proteins to promote the immune response and enhance resolution of precancerous and early cancer lesions.

#### Immunological therapy for HPV

There is considerable research interest in HPV therapeutic vaccine development. During the next 25 years, 5 million women who are already infected with HPV will develop cervical cancer. Vaccines have been developed which appear to work in mice and are immunogenic in humans, but none has reliably halted or reversed cancer progression.

HPV is a nonlytic virus that does not generate local inflammation. The immune response is certainly weaker compared with most other pathogenic viruses.

However, infected cells carry several viral proteins capable of signalling the presence of HPV. The immune system recognises a peptide of 8–10 amino acids on the infected cell surface.

#### CerVax 16

CSL has an experimental therapeutic product, CerVax 16, which uses E6/E7 proteins identical to those of HPV16 and a quillaia saponin-based adjuvant capable of promoting both humoral and cell-mediated immunity [75].

The vaccine has been trialled in a double-blind, dose-escalation study that was stratified by HPV16 status. Participants were 31 women with HSIL smears and, in most cases, CIN3 lesions.

Vaccination induced an E7 specific DTH response and there was a fall in viral load post-vaccination. Anti-E7 antibody developed in all vaccinated subjects and most demonstrated anti-E7-specific T helper cell responses. However, during 12 weeks' followup, there was no change in colposcopy and histology. The women were referred for standard treatment.

#### A therapeutic vaccine for genital warts

An HPV6b VLP vaccine has been trialled in Zhejiang province, China [76]. Women with recurrent genital warts were treated with three doses of VLPs administered at 4 weekly intervals. There was a DTH response at the injection site and a rise in HPV6-specific antibodies. Ten weeks after first immunisation, only 40% of patients still had unresolved warts. A randomised, placebo-controlled study of VLP vaccine as therapy for warts is warranted.

#### Other studies

By 2004, 12 human studies of HPV immunotherapy had been published. In all but one study, the antigen was derived from an E6/E7 fusion protein, but the target diseases varied (genital warts [76, 77], anal/cervical dysplasia [78], cervical cancer, [75, 79–82] VIN [83–85]). Although the vaccines have proven to be well-tolerated and immunogenic, disease regression has been very inconsistent. Some developmental products have been named, and are listed here to aid recognition: Xenova (HPV 16); Stressgen (HPV 16); Zycos (HPV 16).

#### Transplantable tumour models

Grafting and transplanting tumours in animals has demonstrated that effective epithelial immunotherapy requires

- effector CD4 and CD8 T-cells,
- IFN-*γ*, but not Perforin or FasL,
- an adequate “magnitude of response,”
- local inflammation, even when an effective cellular immune response is induced.

#### Conclusions

Therapeutic vaccines to combat HPV infection are at least a decade away. They appear unlikely to be effective as sole therapy for HPV-associated tumours. Enhancing innate immunity may prove to be as important as generating antigen-specific responses.

## VACCINATION AGAINST CERVICAL CANCER: IMPACT ON SCREENING

### Jack Cuzick

#### Cervical screening issues

Cytology is unfeasible for much of the developing world due to its cost, inadequate infrastructure and levels of expertise, and the very high level of inflammatory smears (false-positives). In an example from Recife, Brazil, in 1991, ∼ 63 000 women were screened using Pap smears. The incidence of inflammatory changes was 71%, compared with 6% of CIN or cancerous lesions. Only one in five women recorded “normal” Pap smears.

Because of the long time period over which epithelial dysplasias develop neoplastic tendencies, a programme must operate continuously and consistently over time for greatest impact on mortality rates.

#### Prophylactic vaccines

The introduction of commercial vaccines will raise many strategic questions with regard to cervical cancer prophylaxis and management.

#### Target groups

There is some debate over which groups to target for vaccination, for example, whether it should be available to all women or restricted by age or HPV status. Men, who are a reservoir for HPV virus, may also be considered for vaccination.

#### Efficacy measures

While the ultimate goal of HPV vaccination is to reduce cancer deaths, it will be a long time before that reduction is measurable. Success may also be evaluated through levels of HPV infection, persistence of infection, levels of CIN, or only high-grade CIN.

The durability of protection needs to be evaluated to determine whether boosters will be required to maintain immunity following the initial series.

Neither of two vaccines currently in development targets all HPV types identified as high-risk for cervical cancer. At best, they would only reduce cancers linked to HPVs 16 and 18 (65–75%). To achieve 85% protection, a vaccine would need to be immunogenic against five different high-risk HPVs, yet the relative importance of the various HPV strains in cancer causation differs between regions [35].

The interpretation of diagnostic HPV tests following the introduction of the vaccine will be more difficult. Following vaccination, women would test positive to the HR-HPV screen used in HC II tests. Over time, there will be a need for tests capable of distinguishing HPV types 16 and 18 from other HPV types linked to cervical cancer.

#### Timelines

Within 1–3 years, the impact of vaccination on CIN2+ should be evident. Proven efficacy against cancer is likely to require 5 years, and will probably be seen earliest in the developing world. The effectiveness of vaccination on preventing persistent infection for the types used remains at 100% [86, 87].

Vaccination will not reduce the need for regular cervical screening for at least 10 years, and probably longer, depending on levels of usage, HPV type distribution within the population, and screening techniques used.

## HUMAN PAPILLOMAVIRUS DNA TESTING: WHAT ARE THE PSYCHOSOCIAL ISSUES?

### Marian Pitts

A review of the role of HPV testing within a cervical screening programme identified “a lack of knowledge about the psychosocial issues involved in providing cervical screening in general and HPV testing in particular [69].”

Public support for HPV testing and appropriate infrastructure and technology will need to be available before testing can become generalised; perhaps more important will be general knowledge and understanding.

Of the few studies to examine knowledge of HPV, most have sampled US university students. Both Ramirez et al [88] and Baer et al [89] reported very low levels of awareness of HPV, particularly its link to cervical cancer.

These studies did not compare HPV knowledge with understanding of cervical cancer and screening. Consequently, it is difficult to know whether gaps in the knowledge base are broad or restricted to specific topics.

Pitts and Clarke conducted a study at a UK university in 2002 to examine knowledge of HPV in the context of cervical cancer, and understanding of the screening process [90].

The sample group of 985 women was 19–64 years of age (mean = 40 years). Approximately half worked in clerical or administrative roles; academics, managers, and manual workers accounted for the remainder.

The GP (64.3%) and practice nurse (50.3%) were the most frequently cited sources of information regarding cervical cancer. However, family/friends (30.5%) and magazines/books (29.3%) were also significant.

It was encouraging that when the women were asked what an abnormal smear might mean, 97% of women mentioned abnormal, precancerous cells; 39% mentioned cancer; and 45% mentioned infection. Less than 1% of women said they did not know.

Two in three women (68%) were aware that a large number of sexual partners could increase risk, and 60.3% also mentioned early age of first sexual activity. Smoking (45%) and failure to use condoms (28%) were mentioned by fewer women.

However, only 30% of sampled women were aware of HPV, and among the aware minority, knowledge was generally poor (78% incorrect or no knowledge). Only 30% of respondents were aware that HPV is a sexually transmitted disease, and consequently very few participants could correctly identify risk factors.

In answer to more specific questions, the majority of respondents admitted they “did not know.” For instance, in response to the question “if symptomatic, what are the signs and symptoms of HPV?,” 90% answered incorrectly or left the question blank. As for the long-term effects of HPV, only 11% of respondents demonstrated good understanding.

To determine what women want to know about HPV, Anhang et al reported on eight ethnically diverse focus groups [91].

The women were provided with background information before the focus groups met. In discussion, it was evident that women overestimated the likelihood that HPV exposure would lead to cancer, and struggled to balance this knowledge with the awareness that HPV often regresses without treatment. Consequently, they found it difficult to assess personal risk of HPV and cervical cancer, often failing to understand how a Pap test could be normal in HPV+ women. Younger women focused on sexual transmission of HPV rather than its potential to cause cancer.

Anhang et al paper suggests that without good communication and understanding, responses to a diagnosis of HPV could include anxiety, anger, regret, and fear [91].

#### HPV testing: emotional and psychosexual impacts

Among women who have had a positive HPV test, anxiety about the potential for cancer has been demonstrated repeatedly. McCaffrey et al found that HPV-positive women were more anxious and concerned about relationships compared with HPV-negative women [92].

Although there is no evidence specifically related to HPV, the stigma and concern associated with a positive diagnosis for an STD have been widely reported [93, 94].

#### Role of the media in education

In 111 US newspaper stories (1995–2002) on HPV, there was little information regarding prevention, transmission, and symptoms. Only a minority of stories mentioned risk factors for HPV, stated that HPV can be asymptomatic, or included the frequency of regression without treatment. In fact, only one in four mentioned that most HPV+ women do not develop cervical cancer.

#### HPV among gay men

A survey in Melbourne, Australia, of 384 well-educated gay men found little understanding of anal cancer and the role of HPV [95]. More than half of those interviewed had not heard of an anal Pap smear and/or HPV, suggesting a poor sense of personal susceptibility to HPV disease.

Among those who were aware, the most common source of information had been a doctor or other health professional. The results suggest that health education for gay men should not be neglected.

#### Moving forward

There is a clear need for further studies. Few evaluations have assessed risk perceptions or the likely impact of HPV testing or vaccination on cervical screening.

As HPV testing becomes more widely available, particularly with the advent of vaccines, it will be important to determine how to educate the community in an effective, strategic, and consistent way.

## CERVICAL CANCER SCREENING: WOMEN'S PERCEPTION, PREFERENCES, AND ACCEPTANCE

### Partha Basu

#### Cervical screening in India

Cervical screening is a new concept in India. In order to assess the acceptability of cervical screening, perceptions, and preferences among women, and reasons for noncompliance with screening, a review was undertaken wherein women were offered the opportunity to undergo a free screen.

Screening was undertaken using VIA, VILI, and HCII tests following counselling, and the service was provided in a location close to their homes.

#### Understanding noncompliance

Five hundred randomly selected women who did not attend the screening programme were interviewed by a medical officer. She used a structured questionnaire based upon feedback from a series of focus group discussions.

The questionnaire included 24 potential reasons for non-compliance and was undertaken by 469 women, 61% of whom were illiterate and 75% were housewives. Most came from poor socioeconomic backgrounds (86%) and the majority (61%) were < 40 years of age.

Reasons for nonattendance varied. Among 232 (49.5%) women who were unwilling to attend, 46.1% believed that there was no need for a checkup as they were not sick, while others expressed fears about the cancer detection test (36.2%). Some women felt that they might also experience problems reported by a relative/neighbour following testing (27.6%). Among other responses, the most common was a fatalistic approach to destiny (18.5%).

Over 40% of nonattendees were willing to accept screening but could not attend the clinic due to various reasons, the commonest being work or family commitments. In this group 26.5% of respondents claimed that their husband/in-laws did not allow them to be tested. One in 20 women claimed to have been advised against testing by their doctor.

A small number (5.8%) attended clinic without being tested; some became impatient with waiting, were scared by the instruments or refused to be seen by a male doctor (2.1%).

#### Post-screening feedback

Women (*N* = 498) from 13 randomly selected villages who underwent screening were interviewed by a female social worker. Most reported no pain or only slight discomfort during screening (94.2%). Some experienced post-screening issues such as burning sensation (5.8%), vaginal discharge (12%) or bleeding (3.8%). Seven women subsequently sought medical attention for post-screening problems.

The majority of women were satisfied with the screening service (94.6% selected the top three of six rating options) and 97% said they would recommend the test to others. A small number (18/498) said their husbands were unhappy with screening.

The most common reasons for dissatisfaction with the screening were pain/discomfort during or after screening, long waiting time, failure to address other health complaints, and inadequate explanation regarding followup.

#### Improving screening

Noncompliance remains a major barrier to screening in India, reflecting the absence of a preventative health orientation, and the lack of empowerment of women. This review showed that screening is generally well accepted among women, suggesting it should integrate with primary healthcare.

To maximise the uptake of screening and satisfaction therewith, the interviewed women made the following suggestions.

- Other medical problems should be addressed.
- Medical assistance could be offered to children.
- Medicines should be provided free of charge.
- Female doctors are preferred.
- Men should be included in group counselling meetings.

## CURRENT PROBLEMS FOR CERVICAL CANCER SCREENING IN JAPAN

### Ryo Konno

#### Start of cervical cancer screening

Mass screening for cervical cancer was introduced in Japan in the 1960s. In 1982, the government enacted the Health and Medical Service Law for the Aged, which supported annual screening for all women > 30 years of age. Screening uses the Japanese Society of Obstetricians and Gynaecologist scheme to classify Pap test cytology with minor modification [96].

In Miyagi prefecture [97], the screening rate of the female population aged > 30 years was 0.2% in 1961. Thereafter, it gradually increased, and the screening rate was 30.4% in 1991. The mortality rate due to cervical cancer fell from 12.1 in 1961 to 4.0 in 1994. In Japan, the age-adjusted mortality rate of uterine carcinoma fell from 21.3 in 1960 to 5.3 in 1993. Cervical cancer is the 8th in the rank of cancer deaths in women. The reduction seemed to be due to the spread of screening. A 1998 statement of government endorsed the efficacy of screening, stating that further reductions in mortality would occur [96].

#### Low coverage of screening

However, the Japanese national government stopped funding cancer screening in 1998. At present, the decline in screening rate is a large problem. The annual screening rate of cervical cancer nationwide has fallen since 1993, since the opportunity of appropriate education by government in cervical cancer decreased [98]. In 1997, some mass media with a lack of knowledge claimed that mass screening for cervical cancer might not be effective. Only 22% of women underwent a Pap test in 2002 in Japan, whereas among women aged 18–44 years in the USA, almost 90% had been screened in the previous 3 years [99].

#### Increase in cervical cancer mortality

**Figure 4:**
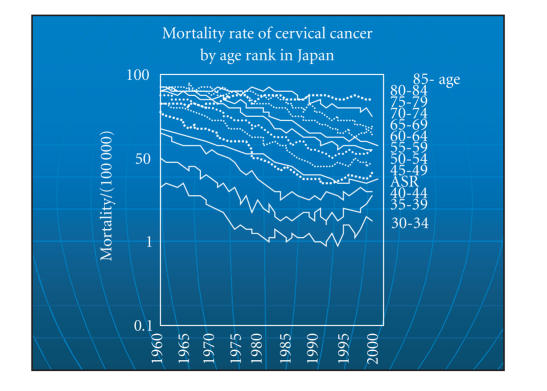
Mortality rate of cervical cancer by age rank in Japan.

In all ages, the mortality had steadily decreased until 1990, but changed to an upward trend after 1995. In terms of age, there is a tendency that the mortality has decreased in the population aged > 50 years but has increased those < 50 years since 1990, and the increase is more remarkable in the younger population (Figure 4) [98].

Recognising some of the problems associated with cervical cancer screening in Japan, the Anticancer Committee of the Japan Association of Obstetrics and Gynecologists appealed for revised legislation. It was proposed that eligibility for screening be extended in an effort to detect cervical cancer earlier, avoid hysterectomies, and allow women to conceive. Legislation was revised in 2005 to initiate biennial screening for women from 20 years of age and to improve education regarding HPV.

#### Screening: knowledge, participation, and motivation

An internet survey of 2000 randomly selected Japanese women (20–59 years of age) was undertaken in April 2005. This was designed to evaluate knowledge of cervical cancer, motivation for participation in screening, and knowledge of papillomavirus and tests for HPV.

Over 1000 (51.9%) questionnaires were available for analysis.

More than half the women surveyed said they knew about cervical cancer and the methods of cervical cancer screening. A similar number (51.3%) were aware that the incidence of cervical cancer had increased in Japan in recent years, yet almost 60% were unaware that the disease was not terminal when detected early and treated.

The most frequently mentioned sources of information were TV/radio, books, and the Internet. Although 49% of respondents said they had been tested for cervical cancer, only 18% were screened annually.

Among women who were not screened, the most common reasons cited were the troublesome nature, cost or shame of the procedure, and inadequate time. Very few women (13%) were aware of HPV or its means of infection.

However, at least three in five (61%) said they would be willing to undertake an HPV test.

One hundred and twenty-six respondents made suggestions as to how screening could be improved. Their demands were mainly for more information and/or funding from the government to reduce the cost of the tests. Some expressed the desire for greater protection of privacy (55 mentioned having a female doctor) and for screening to be easier.

#### Remarks

There is clearly inadequate education and understanding of cervical cancer within the community. Furthermore, there is presently no clear quality direction for the screening programme—Pap testing is not liquid-based, is not classified by the Bethesda system, or backed up with an HPV DNA test. There are no guidelines for SIL management.

Recommendations to improve the system include extending annual screening to women < 30 years of age, with biennial screening for women > 30 years following three consecutive negative tests, or including HPV DNA testing in a triennial screen [100].

Regardless of the method, more education on cervical cancer, screening, and HPV is needed.

## THE PHILIPPINES: TRAINING NEEDS FOR ANTICERVICAL CANCER MEDICAL EDUCATION AND COMMUNITY INFORMATION

### Cecilia Ladines-Llave

#### The Philippines

The Philippines is an archipelago of 7167 islands, with a population of more than 87 million people. It is a young nation by age—only 4% of the population are > 65 years of age. Poverty is widespread, and communication and transport are difficult, particularly in rural areas.

Pap smears cost P400 (US$7). The minimum daily wage is US$3.57 and the average family income is US$221/month. Preventative health is a luxury.

Twenty million women are aged 25–55, the target population for cervical cancer screening. Twenty-three percent of women have experienced sexual activity by age of 24, including 1.2% before age of 13.

#### Cervical cancer burden in the Philippines

Cervical cancer is the Philippines' fourth most prevalent cancer, behind lung, breast, and liver tumours.

In a single year, there are over 4500 new cases of cervical cancer. Most (93.5%) are linked to HPV 16 or 18, and are not detected until they have become invasive. The incidence is likely to be underestimated—systematic data gathering is poor, particularly in rural areas where the majority of the population (62%) lives. Coverage by the Cancer Registry is only 25%.

There is no organised and sustained cervical cancer control programme, and only 12% of the population are screened. Responsibility for such a programme was recently moved from the Department of Health (DOH) to Local Government Units. Unfortunately, these units are overloaded with patients and multitasking reduces their effectiveness.

#### Education and learning

Knowledge about cancer, and particularly cervical cancer, is poor, due to the lack of readily available responsible public information and trained medical personnel.

Among the 3600 general practitioner graduates from 39 medical schools each year, 68% leave for overseas, as do 50% of nursing graduates. Those who do not migrate practice in urban areas, leaving rural areas under the care of inadequately trained health workers. Education regarding cervical screening must extend beyond doctors to include cytotechnologists, gynaecological and oncology nurses, and midwives. In many areas, screening currently relies on *barangay* (public) health workers.

#### Plans for the future

A range of options are under consideration to improve population screening for cervical cancer. Before systems can be expanded dramatically and a public information campaign is initiated, diagnostic and therapeutic facilities must be able to cope with increased demand.

The Cervical Cancer Research Project has been planned as a cooperative venture between local, national, and international organisations. In 2002, a pilot programme began in Cebu province, which is now being used as a training centre for implementation in other provinces. The DOH mandated the implementation of an improved national screening programme in 2005.

A training manual has been developed and guidelines prepared for setting up clinics. The plan includes a directory of key contacts and a registry of the target population.

When diagnostic and therapeutic centres are in place and sustainable, there will be a media campaign to educate women, with ongoing health education to maintain their interest. Advocacy meetings among medical, diagnostic, more general interest groups and support groups, are planned. Solutions have been identified for resource and programme sustainability problems.

## SCREENING EXPERIENCES: VISUAL INSPECTION WITH ACETIC ACID (VIA)

### R. Sankaranarayanan

#### Introduction

Simple and less expensive methods of cervical screening based on visual examination of the uterine cervix are currently being investigated as alternatives in low-resource settings. VIA has been widely evaluated for accuracy in detecting CIN 2/3 lesions in research settings in low-resource countries. Suspicious CIN lesions are characterised by well-defined acetowhite areas in the transformation zone surrounding the cervical os, in close proximity with the squamo-columnar junction, or by acetowhite lesions occupying the entire cervix. The immediate availability of test results following VIA facilitates the diagnosis and treatment of lesions in the same session, which has important logistical advantages and ensures a high participation.

#### Accuracy of VIA

A series of reports on the use of VIA in South Africa [101–103], Zimbabwe [104], Iran [105], Egypt [106], Uganda [107], Cameroun [108], Peru [109], China [110], and India [111–113] suggests that its sensitivity to detect CIN 2/3 lesions ranged from 67–94% and the specificity from 44–96%. Pooled data from several studies indicate that the average sensitivity and specificity of VIA to detect high-grade cervical lesions is around 70% and 80%, respectively. VIA had a similar or higher sensitivity than that of cervical cytology in many studies in developing countries where both the tests were concurrently used, although it had a lower specificity than Pap smear. Three studies that compared VIA with and without magnification provided variable results for specificity and sensitivity, but notably results were fairly consistent within each study, indicating that magnification did not improve the test performance over and above that of naked eye visualisation [101, 113, 114].

#### Findings in randomised trials

The efficacy of a once in a life-time VIA screening in reducing incidence of and mortality from cervical cancer is being assessed in two cluster RTCs in India. In one, women aged 30–59 years in Dindigul district, South India, were randomised to VIA screening by nurses (*n* = 48 225) and to a control group (*n* = 30 167), which received health education and existing care [50, 115]. Of the 30 577 eligible women screened, 2939 (9.6%) VIA-positive women were investigated with colposcopy by nurses and 2777 (9.1%) women had biopsy. The detection rates of lesions per 1000 screened women in this study were 58.2 for CIN 1, 7.3 for CIN 2-3, and 2.3 for invasive cancer. Followup of the study groups is in progress to establish cervical cancer incidence and mortality.

The second trial, involving 130 000 women in Osmanabad district, Western India, investigated the cost-effectiveness of a single round of VIA, cytology, and HPV testing in reducing cervical cancer incidence and mortality as compared to a control group with usual care (no screening) [116]. Of the eligible women, 72–74% were screened. Test positivity rates were 14.0% for VIA, 7.0% for cytology, and 10.3% for HPV. The detection rate of high-grade lesions was similar in all intervention arms (0.7% for VIA, 1.0% for cytology and 0.9% for HPV testing), with 53–67% of invasive cancers diagnosed in the screened groups during stage I as compared to 19% in the control group. The total costs per eligible woman for screening and diagnosis were US$4.5, US$7.3, and US$12.7 with VIA, cytology, and HPV, respectively [117]. Between 10% and 24% of these costs were programmatic, including implementation and management. The cost per CIN 2/3 detected using VIA compared with no screening was $775 (95% CI 678–893); the incremental cost of cytology compared to VIA was US$1135 (95% CI 794–1958) per CIN2/3 detected [117]. The ultimate effectiveness of the three approaches will become clear with followup for cancer incidence and mortality.

#### Conclusions

Studies indicate that women are willing to participate in and accept VIA screening, and detection rates of early lesions using visual testing attest to its validity and usefulness. Good training is needed to achieve fairly accurate and moderately reproducible results with visual tests in developing countries. A wide range of health care providers including trained medical and nonmedical personnel can provide VIA after roughly 5–10 days of competency-based training.

Quality assurance of visual screening in field conditions poses a major challenge. Close monitoring of test positivity and disease detection rates as well as periodic retraining are essential to maintain good standards of visual testing. While a resultant reduction in the incidence and mortality associated with cervical cancer following VIA screening has not yet been proven, this is currently being addressed in randomised screening trials.

## CERVICAL CANCER SCREENING USING A COMBINATION OF PAP AND DNA TESTS

### Masaki Inoue

#### The screening programme in Japan

A nationwide cancer prevention programme using annual Pap smears has dramatically reduced cervical cancer incidence and resultant mortality in Japan. However, the Pap test is not always accurate, and false-negative results can have serious implications. This has led to a reevaluation of the cancer screening programme in Japan.

#### Pap with HC II

A recent study evaluated a combination of Pap smear and HPV DNA testing for routine cervical cancer screening. The focus was on Ishikawa prefecture, in the northwest of Japan's main island. Kanazawa is the capital of this prefecture, which has a population of 1.2 million.

More than 8000 women were recruited into the study between October 2003 and April 2004. Two cytology samples were taken from the ectocervix using a cytobrush, one for Pap smear and the other for HPV DNA test. HC II for high-risk types of papillomaviruses was used, and positive samples were classified further using the DNA-chip method.

Cytological diagnosis was according to the classification system established by the Japanese Society for Obstetrics and Gynecology (JSOG). This differs from the Bethesda system in classification terminology, but classifications IIIa, IIIb, IV, and V would equate to the ASCUS, LSIL, HSIL, and invasive cancer categories. Colposcopy and biopsy were recommended in all cases catalogued above JSOG II.

HPV was detected in 7% of women with normal cytology. Overall incidence of HPV infection was 11%. It was more common for younger women to test positive for HPV than older women, with 45% of women aged 15–19 years testing positive. More than 1500 women in their 20s were tested—24% were positive for high-risk HPV. In women > 40 years of age, levels of infection fell to 4–7%.

There was a higher incidence of Class IIIa cytology among HPV+ women (74%) than among HPV− women (41%). Among 26 women whose cytology was normal, but who had CIN lesions on histology, 23 (88%) tested HPV+. Among 57 women with abnormal cytology, 86% had CIN lesions and 92% were HPV+. This demonstrated the potential for HPV testing to detect women graded normal with routine cytological screening, despite serious lesions (CIN3 or invasive cancer).

#### Typing HPV in Ishikawa precinct

Those samples that were positive with hybrid capture assay were further examined using Biomed-Lab DNA-Chip. The most frequently detected type of HPV in these Japanese women was HPV 52, followed by HPV 16.

Almost half the samples were infected with more than a single HPV type. Multiple infection was more common among younger women, particularly those in their late teens.

It was more common to find multiple infections in women with CIN lesions than in women with invasive cancer.

In 2004, as a result of these findings, Japan introduced a new approach to screening for cervical cancer. Samples with questionable cytology (ASCUS using the Bethesda classification system) are routinely examined for HPV DNA. HPV+ women are then recalled for colposcopy.

As in this sample from Kanazawa city, it is clear that the use of HPV testing has improved the positive predictive value of cervical cancer screening. Among 400 samples that revealed questionable cytology, 50 cases were HPV+ on testing, so an additional 42 women were referred to colposcopy. This detected an additional four cases of CIN2 lesions and two of CIN3 lesions. These six women were treated surgically—earlier than if the HPV test had not been used.

#### Changing screening policies

In conclusion, the combination of Pap cytology and HPV tests has improved cancer screening efficacy.

The HPV DNA test incurs additional costs. However, for women who are negative on cytology and HPV, the inter-screening interval could be extended, thereby recouping the cost.

## THAILAND: CERVICAL CANCER SCREENING AND EPIDEMIOLOGY

### Somkeart Srisupundit

#### Cancer in Thailand

Data from 1996 showed that cervical cancer was the most common cancer in Thailand women, with an ASR of 19.5, higher than that for breast cancer (ASR ∼ 17.2) [118].

Within Thailand, incidence varies between regions, with Chiang Mai (ASR 25.6) and Lampang (23.6) having higher than national levels. The age prevalence is fairly consistent between regions, peaking at 45–55 years of age.

Unlike more economically developed countries, where most cervical carcinomas are detected early, only one in five Thai cancers will be detected in Stage 1 [119].

In 2005, the cervical cancer incidence was 19.5/100 000 women. This equates to 30 000 cases of invasive cancer over a 5-year period. Five women die from cervical cancer every day in Thailand, and 10 new cases are detected daily.

#### Screening for Thailand

Thailand has limited resources for screening. In 2004, a cytology-based programme was established, which recommended screening every 5 years and aimed to achieve 50% coverage. The screens were most commonly undertaken at family planning clinics.

By 2005, the programme was demonstrating coverage rates of only 10–15%. The lack of cytoscreeners and pathologists led to long delays in receiving test results, and no firm policy had been established to assist healthcare professionals to deal with abnormal results.

#### Training and treatment

An intensive and thorough training programme has now begun to build competency in VIA and cryotherapy.

Clinics are encouraged to rely on VIA if Pap smears are not performed efficiently. Ten of the 75 Thai provinces have adopted this approach. Women 30–45 years of age are encouraged to be screened every 5 years. The objective is to achieve 80% coverage over 5 years.

In Roi Et Province, a concerted effort has been made to deliver the screening service to women. Mobile units visit rural health centres. With these intensive mobile units, district teams are able to test three times as many women in a week as services at district hospitals. This success has demonstrated the effectiveness of taking the service to the women, rather than relying on women to attend screening at a distant location.

Health centres staffed by 3–5 persons are at the core of Thailand's primary care system. They generally provide approximately 5000 residents from 8–12 villages with basic medical care and health prevention and promotion programmes.

Involvement of the health centre staff in cervical cancer prevention activities is essential. They identify the target population by visiting all community households and build a registry of names of women in the 30–45-year target age group, to track their attendance at clinics.

The community health centre takes responsibility for organising, promoting, and coordinating designated cervical screening days. Staff will inform the local population about the VIA test using loudspeakers, letters, and presentations. Health volunteers in each village also contact women directly.

This programme has proved being more reliable and efficient than any other, and is being expanded to additional districts and provinces.

## KOREA: CERVICAL CANCER SCREENING AND EPIDEMIOLOGY

### Hai Rim Shin

#### Cervical cancer in South Korea

Since 1980, Korea has had a national tracking system for major cancers, which includes site-specific and regional tracking. The database for national cancer incidence is estimated to be 95% complete.

Cervical cancer is the fourth most common cancer in Korean women. With an AAR of 15.5 in 2001, it is less common than cancers of the stomach, breast, and colon/rectum.

**Figure 5:**
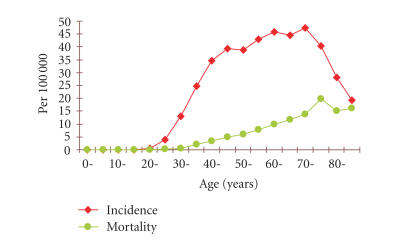
Age-specific incidence and mortality for cervical cancer in Korea.

Between 1993 and 2001, the curves for age-specific incidence of uterine cancer fell, with this trend most marked among women 50–70 years of age (Figure 5).

Almost 40% of malignant cervical cases are being detected in situ, before they become invasive. For women diagnosed with cervical cancer during the period 1995–2001, 6.6% died within 12 months. Almost 80% survived for at least 5 years.

There are local variations in the incidence and mortality of cervical cancer. Morphologically, the majority of tumours (∼ 80%) are of squamous cell origin; however, during the 10 years from 1993, there was a growing proportion of adenocarcinomas (6.9% in 1993; 10.4% in 2001). The 5-year survival rate for adenocarcinomas (74.3%) is less than that for SCC (81.4%).

#### Epidemiology and risk factors

Key factors associated with a lower risk of cervical cancer in Korean women are [120]

- no family history,
- no/few children,
- marriage,
- late first sex,
- circumcision.

In a review of HPV types in Busan for the IARC HPV International Prevalence Survey, the seropositivity of antibodies to HR-HPV (types 16, 18, 31, 33, and 58) was 19.8% among sexually active women 20–74 years of age. The most common types were HPV 70, HPV 33, and HPV 16 [121]. All seven women with HSIL lesions on cytology tested positive for HR-HPV.

#### Busan HPV prevalence study in young women

This trial was undertaken by the Korea National Cancer Center with the Unit of Field and Intervention Studies in the International Agency for Research on Cancer, in 2002, to determine at what age women become sexually active, exposed to HPV-DNA, and then seropositive for HPV 16 and HPV 18. It also provided an opportunity to assess willingness to participate in future HPV vaccine trials.

Students 16–26 years of age attended a health education class and were asked to complete a survey. They provided a blood sample and either a vaginal (self-collected) or penile (physician-collected) swab.

Among 672 females, 15.2% tested positive to HPV, 9.4% of whom tested positive to a high-risk type. Among this latter group, there was a high frequency of infection with multiple strains. Among males 8.7% tested positive for HPV [122]. The risk for infection clearly rose with the number of lifetime sexual partners.

Among this group of 1100 university students, 64% of men and 58% of women said they would be willing to participate in a trial for HPV vaccines.

#### Cervical cancer screening in Korea

Since 1988, medical insurance beneficiaries have received a Pap smear for cervical cancer check as part of their general health checkup. In 1999, the National Screening Program began covering Medicaid patients' screens for stomach, breast, and cervical cancers. Since this time, coverage has been expanded and about 50% of insured patients are currently included.

The current recommendation is that all women > 30 years of age should have a Pap smear every 2 years. Participation in cervical screening in 2002 was 15.6% and 10.8% in 2003. Incorporation of HPV testing into the screening process may improve sensitivity and allow the screening interval for many women to be extended.

## PREVALENCE AND IMPACT OF CERVICAL HPV INFECTIONS IN TAIWAN

### Tang-Yuan Chu

#### Taiwan's national cervical screening programme

In 1988–1992, the AAR of cervical cancer in Taiwan (22.2/100 000) was higher compared with other Asian nations with Chinese populations, such as Singapore (16.3/100 000) and Hong Kong (15.3/100 000).

In 1995, Taiwan instituted the National Cervical Screening Program for women aged > 30 years.

As part of the programme, extensive and ongoing training has been introduced for cytotechnicians, nurses who collect samples, and gynaecologists undertaking colposcopy. To participate in the programme, laboratories and their staff must be accredited and are reviewed regularly.

Every year since 2000, more than 50% of eligible women had participated in cervical screening within the previous 3 years. From 9.2% of eligible Taiwanese women screened in 1995, screening levels rose annually until 2001, when 30.1% of women undertook a screening test; 27.1% of Taiwanese women were screened in 2003.

When the programme began, cervical cancer levels appeared to rise from 30–35/100 000 in 1995 to over 50 in 2000. This probably reflected more frequent diagnosis due to increased screening levels, rather than greater incidence. Furthermore, the AAIR peaked in 1999 and declined in the next 2 years. During the same time period, the proportion of cancers that were detected in situ relative to invasive cancers rose dramatically, reflecting earlier diagnosis of the condition.

#### HPV and cervical cancer in Taiwan

In 1992, a nationwide survey of more than 13 000 women was undertaken across seven counties and reported by Liaw et al [123]. This demonstrated the very high incidence of HPV infection among woman showing cervical abnormalities. Among women without lesions, the incidence of HPV infection was 9%; among 40 women with CIN1 changes, HPV incidence was 54%; and among those with CIN2, CIN3, and invasive cancer (*n* = 48), the incidence of HPV infection was 92%. HPV strains 52 and 58 were the most commonly detected.

Hsu et al reported the results of a population screen for HPV virus using MY09/11 PCR and sequencing [124]. Among women > 55 years of age, the incidence of HPV infection was 19.4%. Age-specific data from 1999 demonstrate the highest levels of confirmed cervical cancer among women aged 70–74 years, an age where incidence peaked at over 180/100 000 women.

The link between infection with HPV and the development of cervical cancer is also reflected in other results. Patients who showed SIL on Pap test were eligible for the study; 1284 women were recruited. Infection with HPV was confirmed using HCII and PCR-Strip testing. Among those with low-grade lesions, the incidence of HPV infection was 75%. Those with high-grade lesions had an HPV incidence of 83.6%. All (100%) 16 women with invasive carcinoma tested positive for HPV.

Further research has evaluated the relative risk of cervical cancer for different strains of HPV. More than half of women (51%) with cervical SCC tested positive for HPV 16, while HPVs 18, 58, and 33 were also frequently detected. In cervical adenocarcinoma or adenosquamous carcinomas, HPV 18 was detected in 58.8% and HPV 16 in 35.3% of affected women. Among 263 cases of cervical cancer in Taiwan, the majority were linked to five strains of HPV: 16 (50.7%); 18 (11.9%); 58 (10.1%); 33 (8.4%) and 52 (3.1%).

ASCUS and AGUS abnormalities detected cytologically, and frequently associated with venereal warts, were also evaluated for HPV status (*n* = 436). Strains of HPV most frequently associated with ASCUS and AGUS cytology were 52 (18%), 16 (15%), and 58 (15%).

Among the Taiwanese population, HPV types identified as high risk for cervical cancer are most prevalent, occurring at more than twice the frequency of all other types of HPV. In a survey of 4190 people who tested positive for HPV, the most common types were 52 (22.3%), 16 (11.7%), and 58 (11.2%). Low-risk types were detected in 18.4% of cases.

Compared with other countries, Taiwan has a disproportionately high incidence of HPVs 52 and 58. This dramatically contrasts with Europe, where HPV 16 is the predominant strain. Phylogenetic trees show that differences in the origins of HPVs 58 and 52 can be traced in Asian nations such as Thailand, Hong Kong, and Taiwan.

#### Ongoing development of Taiwan's screening programme

Pilot studies are now underway to investigate the most cost-effective way to include routine HPV testing in Taiwan's cervical screening programme. A self-screening test using menstruation pads is among the options being investigated.

Due to the extensive recording undertaken for the screening programme, Taiwan has data to conduct simulation modelling, which can aid planning decisions. The country has also been involved in trials for HPV vaccines currently in development. In the short-term, decisions will be made regarding frequency of testing (possibly extended to 5 years) and the timing of HPV testing relative to smears and cytology.

Taiwan has already experienced success with a vaccination to prevent cancer. Following the launch of mass vaccination against hepatitis B, incidence of hepatocellular carcinoma dropped significantly.

As the majority of cervical cancers can be linked to infection with a few high-risk strains of HPV, vaccination offers significant potential to reduce the incidence of cervical cancer in Taiwan.

## CLINICAL ALGORITHMS FOR CIN1

### Laurie Elit

#### Evidence-based approach to LSIL cytology smears

Emerging evidence has changed the way low-grade cytological changes are viewed, but it has not clarified patient management. Members of the Ontario Cervical Screening Program reviewed the available evidence in order to optimise national guidelines.

The interdisciplinary guideline committee defined a series of questions regarding the cervical screening process, with the aim of defining the optimal management for women with abnormal cytology (up to, but not including, colposcopy).

The Appraisal of Guidelines, Research, and Education (AGREE) process was chosen as the framework for reviewing the guidelines. AGREE consists of 23 Likert-scale items organised into six domains, and offers confidence that potential biases in guideline development have been addressed.

#### Review of existing guidelines

The guideline search and retrieval included some online databases, known guideline developer websites (e.g., http://www.guideline.gov and http://www.g-i-n.net), systematic review websites (e.g., http://www.cochrane.org and http://www.who.int), and references cited on other materials.

Materials specific for LSIL included published guidelines from the American Society for Colposcopy and Cervical Pathology (ASCCP), 2001 [125], and National Health and Medical Research Council (NHMRC), 2004 [126]; one RCT; one meta-analysis; four retrospective studies; and one conference report.

Each source was subsequently evaluated by the panel according to AGREE classifications covering quality, currency, and content.

The two existing guidelines were subjected to detailed review, which considered their scope, stakeholders, rigour, clarity, applicability, and editorial independence.

Every recommendation within the guidelines was reviewed according to the level of evidence on which it had been based. Two different ranking systems were used in this process (Table 6).

#### LSIL cytology

Low-grade lesions present a dilemma when detected during screening. Half of the women with LSIL cytology will be normal if checked subsequently after 4–6 months, yet the remainder will progress to HSIL or invasive cancer within 2 years [127].

Three management approaches could be taken for these patients:

- no action, but repeat cytology in 4–6 months,
- immediate colposcopy,
- HPV testing.

The ASCUS/LSIL Triage Study (ALTS) Group evaluated 5000 women to determine how best to manage these early, inconclusive lesions. They found that HPV testing was likely to be positive and would not clarify management.

The ASCCP Guidelines, based on Level CIII evidence, permit multiple paths following LSIL cytology, with different recommendations for teens and postmenopausal women.

Moscicki suggested further revisions to approaches for adolescents. He believed that LSIL reflected an HPV infection, and commented that “most guidelines in the USA were overaggressive in their management of abnormal cytology in adolescents and young women (resulting in) costly over-treatment” [128]. The median time for HPV clearance is 8–14 months. A repeat Pap smear 12 months after the LSIL finding would allow for 20–25% of cases to regress.

The NHMRC based their 2004 recommendations on Level CII evidence. If a woman is > 30 years of age and has not experienced negative cytology in the previous 2-3 years, she should have immediate colposcopy or repeat cytology after 6 months. If the woman is on biennial surveillance or < 30 years of age with no cervical cytology history, repeat cytology screening in 12 months. A woman should be referred for colposcopy following two abnormal tests; if the second test is normal, it should be repeated after 12 months.

#### Adapting the evidence for Canada [129]

The guideline panel then needed to decide which guideline/s would best meet Canada's needs and goals.

The Program in Evidence-Based Medicine recommended that women with LSIL on Pap smear should be sent for colposcopy or repeat cytology after 6 months (B-II). All women whose cytology is abnormal on the second test should be referred for colposcopy. If the second Pap test is normal, it should be repeated again after a further 6 months and the woman referred if the result is ASCUS or abnormal (C-III).

#### Seeking feedback

Surveys were completed by 180 physicians to review the guideline proposal. Approximately half agreed that patient outcomes would be improved by the proposed changes. However, there was universal agreement that referring all LSIL cases for colposcopy would be impractical.

#### Endorsing, launching, and adopting the guidelines

After deciding which people and organisations should endorse the document, plans were made to launch the revised guidelines in September 2005. Following publication, the document will be reviewed annually.

#### Managing an abnormal (CIN1) biopsy

Evidence was reviewed to assess different management approaches for women who have low-grade neoplasia on biopsy (CIN1). Available evidence did not suggest whether it would be better to followup the finding at a later date or to treat the lesion immediately.

Treatment decisions were based on histological results. However, there was huge variability in how operators coded their findings. False-positives and uncertainty lead to unnecessary investigations, possible over-treatment, anxiety, and increased cost.

A number of investigative immunochemistry techniques offer new insights and potentially more objective foundations for clinical management.

Among the most promising is p16(INK)4a, a protein marker that is only present in cases of cancer, CIN 2/3, and CIN1 lesions associated with HR-HPV [130, 131].

As part of a randomised trial in the best management strategy for biopsy proven CIN1, cone biopsies (*n* = 194) were reviewed by five pathologists and coded [130]. Although there was variability between the pathologists, p16(INK)4a testing reduced the number of false-negative and false-positive results.

A study was undertaken to determine whether p16(INK)4a or other adjuvant techniques might enhance the evaluation of CIN1 (LSIL) lesions [132]. Following the classification of cervical biopsies as CIN1, women were tested for HR-HPV using HCII and separately for p16(INK)4a. Five pathologists reviewed the blocks.

Histopathologic diagnosis was most confused when rating a sample as either CIN1 or normal/reactive. The inconclusive samples were more frequently positive for HPV than for p16(INK)4a.

However, 23% of benign cases were also positive for p16(INK)4a, and half of these were positive for HPV. Ongoing monitoring will reveal the role of p16(INK)4a in these patients' disease courses.

A substantial number of CIN1 patients were negative for p16(INK)4a. Whether p16(INK)4a-positivity has significance in terms of natural history requires ongoing followup of these cases.

## CLINICAL ALGORITHMS FOR HSIL

### Michael Quinn

#### Management of HSIL

Approaches to treatment for HSIL depend upon the type of histology (squamous, glandular, mixed), the size and accessibility of the lesion, the patient's stage of life and the surgical options available.

#### Squamous HSIL management

If the whole lesion is not visualised as it involves the endocervical canal, a loop or cone biopsy should be taken. If it is in range, the lesion can be removed by loop, cone, or surgical ablation.

If microinvasion or ESI are suspected, the margins on the biopsy will be important in guiding management. A cold knife is less likely to denature cells than a loop, and is therefore preferred for reviewing margins.

In the case of noninvasive HSIL, either procedure can be used. There is limited guidance on what endpoint determines a successful outcome. Rate of recurrence of CIN is perhaps the best measure to guide followup.

A Cochrane review reported on 28 trials using different surgical techniques to manage CIN without identifying any “obviously superior technique [23].”

The LEEP approach has been linked to a number of obstetric difficulties. A systemic review that factored in smoking, concluded that following LEEP, women were more likely to give birth preterm (OR 1.81; *P* = .006), but there was no difference in likelihood of caesarean delivery, precipitous labour, induction or neonatal intensive care admission [133].

A retrospective cohort study in Nova Scotia, Canada, by Samson et al reported similar results—LEEP is associated with an increased risk of preterm delivery, PROM, and LBW infants in subsequent pregnancies [134].

A New Zealand study concluded that LEEP and laser cone treatments were associated with significantly increased risk of PROM [135]. The risk increased with cone length.

Studies at the Royal Women's Hospital, Carlton, Victoria, Canada, indicate that dysplasia itself is associated with prematurity and that all excisional methods increase this risk.

#### Microinvasive carcinoma: a disease of screened populations

The FIGO classification system for microinvasive carcinoma has been used since 1995. FIGO Stage 1A tumours are superficially invasive and can only be diagnosed microscopically. They are differentiated by depth of invasion and horizontal extension. No consideration is given to vascular space involvement or to special invasion patterns.

To date, no RCTs have been published comparing management approaches with outcomes in microinvasive carcinoma.

When classifying biopsies, processing is critical. Consideration should be given to how the specimen has been prepared—radial versus sagittal sections, whole specimen, special stains, and so forth.

In distinguishing ESI from CIN 3, if the margins on a specimen are clear, there is no indication for hysterectomy. Seven reports include results on the use of cone resection alone for ESI. Among 255 cases of FIGO Stage IA_1_ tumours, there were six recurrences; of 18 cases of Stage IA_2_, there were three recurrences [136–138].

Östör and Rome reported on long-term outcomes in a series of women treated for microinvasive carcinoma when nodes were positive. Twenty-three patients subsequently died in spite of treatment—those women generally had deeper lesions (3–5 mm) [138].

Rome and Brown were able to demonstrate the anticipated higher risk of recurrence in patients with < 3 mm invasion where LVSI was identified pathologically [139]. Relative risk of recurrence for their sample of 131 LVS-positive women was 7.42 (CI = 2.36–22.61). For 92 women for whom the area of invasion was 3–5 mm with LVSI, the relative risk rose to 22.25 (CI = 5.9–57.96) compared with those who were LVS-negative.

At the Royal Women's Hospital (RWH) in Melbourne, Australia, disease management recommendations for microinvasive lesions are as follows.

- If 1–3 mm invasion, treat as for ESI, unless LVS is seen. Consider hysterectomy in postmenopausal women and when childbearing is complete.
- When the invasive zone is 3–5 mm, a hysterectomy is preferred; however, where fertility is an issue, a cone resection including lymph nodes is an option.
- In cases where the margins of the biopsy are positive, and the invasive depth ≤ 3 mm, the cone may be repeated and retested. If CIN is present at the ecto/endocervix, follow with colposcopy/cytology and ECC.

#### Adenocarcinoma in situ

AIS is a recognisable precursor to invasive cancer. It is frequently multifocal and often associated with squamous lesions. The predictive value of cytology is 75%. Since the 1970s, the incidence of AIS (relative and absolute) has increased; HPV 18 and oral contraceptives have been implicated.

Soutter et al reported retrospective outcomes for 53 women in England, whose AIS was treated with cone alone [140]. Ten percent had early invasive lesions detected on second cone or hysterectomy. At 4 years, the cumulative incidence of hysterectomy was 21%, so long-term surveillance is needed postsurgery.

#### Microadenocarcinoma

This classification includes glandular carcinomas such as endocervical, villoglandular, intestinal, endometroid, clear cell, and adenosquamous.

Östör and Rome reported outcomes for 436 women with microinvasive adenocarcinoma defined as invasion ≤ 5 mm, and associated with complete obliteration of normal endocervical crypts, extension beyond normal glandular fields, and stromal response [141]. Of these women, 126 underwent radical hysterectomy, not necessarily with simultaneous removal of adnexa. There were 15 recurrences and six deaths from the disease.

Another report reviewed retrospective data and recommended that, where invasion was < 5 mm, the likelihood of lymph node metastasis and disease recurrence were very small, and therefore conservative surgery could be considered with some confidence [142].

With the limited data available, the RWH makes the following recommendations.

- Conisation is a good approach when lesions are < 3 mm.
- Lymphadenectomy is recommended whenever LVS is positive.
- Recone if there is any uncertainty.
- For lesions 3–5 mm, a simple hysterectomy is recommended.

#### Summary

Adequate data are available to make recommendations for squamous lesions with confidence. It is likely that adenocarcinomas can be managed similarly, although more data are needed to provide statistical validation.

## GENITAL WARTS: TREATMENT MODALITIES

### Suzanne Garland

#### Genital HPV infection

Genital warts (*condylomata acuminata*) is one of the most common STIs worldwide, usually resulting from infection with HPV types 6 and 11 [143].

The highest rates of genital HPV infection are in sexually active women < 25 years of age. Highest incidence for both genders is between 18 and 28 years of age [144].

In developed countries, genital HPV infection has increased steadily since the 1950s [144]. About 1% of all sexually active adults have had or currently have genital warts [143, 144].

#### Treating genital warts

There are many ways to remove warts (cryotherapy, diathermy/laser ablation, surgical incision, or with trichloracetic acid), but recurrences are common (30–60%). Antiproliferative agents such as podophyllotoxin or 5-FU will cause the warts to regress.

There is no specific antiviral agent for HPV. However, the immune modulator imiquimod, which has specific but broad antiviral activities, has been an approved therapy specifically for genital warts and has been endorsed in US-CDC [145], Latin American [146], European [147], and Australian [148] antimicrobial guidelines. The cream is a patient-applied treatment and used on the affected areas, three times a week.

#### Imiquimod: mode of action

Imiquimod induces interferon-alpha in an early, nonspecific, innate immune response as well as stimulating CMI [149, 150]. Its antiviral activity mimics the natural immune response and limits production of HPV; its antiproliferative activity slows growth of infected keratinocytes.

Activated dendritic cells engulf HPV from infected cells and process the viral antigens on their cell surface [151]. They leave the skin via draining lymphatic channels. When they meet and interact with T-cells that have the specific receptor for HPV antigens, the T-cells begin to divide and migrate from the lymph node to the bloodstream [149, 150]. Once activated, these HPV-specific T-cells express the adhesion molecule antigens LFA-1 and VLA-4 on their surface.

Stimulated by the presence of cytokines and chemokines, T-cells and monocytes migrate toward the site of infection. The T-cells invade the wart and kill infected cells, which are then phagocytosed.

The wart gradually clears and immune memory is established. This can reduce recurrences, as newly infected keratinocytes will be rapidly killed.

#### Using imiquimod: clinical trials

A 1998 study reported the outcomes of an intent-to-treat RCT of self-administered imiquimod cream in 311 patients with external anogenital warts [152]. The cream was administered three times per week until clearance, or for a maximum of 16 weeks. Followup was for an additional 12 weeks. Among patients using placebo creams, 11% of patients' warts resolved spontaneously. Among those using imiquimod, total clearance was achieved in 72% of women and 33% of men (*P* < .0001). If the endpoint was > 50% wart reduction, overall success was 76% for imiquimod patients compared with 28% for placebo. Erythema at the wart site occurred in two-thirds of patients using imiquimod cream, while smaller numbers experienced erosion (32%), excoriation (24%), oedema (16%), and scabbing (15%). Interestingly, erythema was reported by one in four who received the placebo cream containing no active ingredient.

Another study sought to determine whether subgroup demographics influenced treatment outcome [153]. The conclusion was that imiquimod provides a significant benefit independent of gender, initial wart area, duration of current outbreak of warts, previous wart treatment, or tobacco use. However, clearance rate was influenced by circumcision—it was ∼33% higher in uncircumcised males. Thrice weekly application was found to be a slightly more efficacious regime than daily application, achieving total clearance in 62% of participants. Furthermore, there were fewer reports of adverse events. Erythema, erosion, flaking, or ulceration were common.

Patients around the world who had previously been treated using cryotherapy or podophyllin rated imiquimod superior with regard to its convenience and lack of pain [154].

A series of trials also demonstrated the efficacy of imiquimod as an adjunct to ablation (post-laser ablation [155], combined with surgery [156], following surgery to remove anal canal condyloma [157]). It was generally well tolerated and reduced recurrence rate.

## CERVICAL CANCER: RECOMMENDATIONS OF IARC

### Albert Singer

#### New IARC handbook of cervical cancer screening

The IARC Handbook of Cancer Prevention, Volume 10, was launched following a meeting in Lyon, France, in April 2004.

The Working Group of the IARC concluded that screening for cervical cancer by Pap smear effectively prevents mortality from the disease. However, in order for a cytology screening programme to reduce death optimally, it must be well organised and have quality control at every step throughout the process. If these prerequisites are met, an estimated 80% reduction in mortality can be achieved.

#### HPV DNA testing

The Group also concluded that sufficient evidence supports the efficacy of the HPV DNA test in reducing mortality from cervical cancer, which is a rare outcome of HPV infection. Tests for the presence of viral DNA in blood sample can signal potentially precancerous lesions. The identification of the role of HPV in the aetiology of cervical cancer has opened new avenues to preventing the disease by screening and vaccination. The major challenge lies in developing a simple, reliable and affordable test that can be used around the world.

HPV DNA testing is yet to be widely adopted within screening programmes. The suitability of HPV as the primary screening test will be influenced by public acceptability and cost. As many infections are transient, HPV screening below 30 years of age is not recommended. HPV testing can be used in combination with Pap smears until long-term data on its effectiveness become available.

#### Screening in developing countries

As much of the cervical cancer disease burden lies in the developing world, effective screening methods need to be applicable in low-resource settings. VIA or VILI is being considered as a primary screening test by many countries, although there is still insufficient evidence of its efficacy in reducing mortality at the epidemiological level. The validity of the diagnosis depends on the training and skill of the examining doctors.

#### Frequency of screening

An organised screening programme should cover women aged 25–65 years. The IARC advises that annual smears are unnecessary, even with conventional cytology. Screening of women < 25 years of age offers minimal benefit. For women 25–49 years of age, three-yearly Pap smears are recommended, or five-yearly where resources are limited. Five-yearly smears from 50–65 years of age are recommended; screening can cease after 65 years of age, provided there are no suspicious results in the previous two tests (Table 7).

As women with HIV are at higher risk of persistent HPV infections, they should be screened frequently from a younger age.

The need for consistency and rapid turnaround in cytology is a difficult challenge for developing countries. A screening programme based on cytology will be successful only when trained staff can provide efficient diagnosis, and resources are available to investigate and treat abnormal results. It is hoped that ongoing research will make low-cost, low technology screening methods viable options for cervical cancer prevention in these settings.

#### New developments in screening

LBC and semi-automation may improve outcomes of cytology screening, depending on local feasibility. Long-term evaluation and quality control are needed.

New commercial systems based on mRNA require rigorous evaluation before they can be adopted for widespread clinical use. Furthermore, education for all health professionals involved in cervical cancer screening will be needed.

## PRACTICAL COLPOSCOPY

### Albert Singer

#### Genital HPV infection

To improve colposcopy practice, it is necessary to consider the accuracy of colposcopy and biases that can arise. Studies of diagnostic and screening colposcopy provide insights regarding the validity of visual signs, their reproducibility, quality control, and how the process is taught.

Those undertaking colposcopy are usually aware that they are dealing with women whose smears were abnormal, a population with a higher probability of disease; this predisposition to look for disease may lead to bias.

Descriptive terms used to define abnormal colposcopic findings may be divided into those considered to be major changes and others. Dense acetowhite epithelium, coarse mosaic, coarse punctuation, iodine negativity, and atypical vessels are all believed to be major changes.

#### Accuracy and reproducibility

A number of studies have evaluated colposcopy's ability to differentiate normal from abnormal lesions. Mitchell et al reported on a meta-analysis, which concluded that the weighted sensitivity of colposcopy to differentiate normality from all other cervical abnormalities was 96% and specificity was 48% [158].

In differentiating low-grade (normal/LSIL) and high-grade (HSIL/cancer), colposcopy scored 85% for sensitivity and 69% for specificity.

To confirm the efficacy of lesion ablation following colposcopy without histological confirmation, Belinson et al team reviewed 1997 women in rural China [57]. Key factors in determining efficacy included the quality of the light source; adequacy of the operators; cervical characteristics such as the presence of inflammation; and diagnostic thresholds. In another study involving ∼ 8500 women in the same Chinese province, Belinson et al found that self-sampling for HPV DNA detection was less sensitive for CIN > 2 than the direct cervical sample for HPV DNA, but similar to LBC [58].

A number of published studies have compared colposcopy with other techniques to evaluate its reliability in screening; conclusions vary dramatically and are clearly influenced by study design [159–162].

#### Colposcopic signs and significance

Reid and Scalzi developed a scoring system based on sharpness of a lesion's margins alongside colour, vascularisation, and iodine staining to rate colposcopic findings [163]. Carriero et al found this ranking system to be 86.6% effective in correctly rating LSIL lesions [164]. A prospective study by Da Forno et al demonstrated the relative significance of acetowhite colouration, borders and abnormal vessels in colposcopic definitions of “normality [165].”

Pretorius et al recognised that the most likely reason for colposcopists to overlook malignant lesions was their size [166]. When a lesion/lesions extended across more than one quadrant they were less likely to be under-rated.

#### Thresholds of uncertainty

“Assuming colposcopists use the same definitions, reproducibility of colposcopic assessment depends in part on colposcopists using similar “thresholds of certainty” for categorization findings as to normal versus abnormality and grade.” (IARC 2005)

Digital photographic databases can retain data for post-treatment audit and comparison between units.

Education is key to quality colposcopy. Consistency, and consequently quality, can only be maintained if practitioners are taught via rigorous training programmes to adhere to guidelines, and are regularly accredited.

## THE ROLE OF MALES IN TRANSMISSION OF HPV INFECTIONS AND CERVICAL CANCER RISK

### Xavier Bosch

#### Male sexual behaviour

Over the years, both social and medical researchers have found statistical links between observed behaviours and the incidence of cervical cancer. As early as the 1850s, differences in incidence were noted between prostitutes and virgins/nuns. During the following century, risk factors for cervical cancer was related to women whose husbands

- travelled as part of their occupation,
- indulged in extramarital sex or multiple marriages,
- had cancer of the penis,
- had a previous wife who had cervical cancer,
- were uncircumcised.

The discovery of HPV as a sexually transmitted carcinogen provided an explanatory context for these observations.

#### IARC studies

Spain (AAIR for cervical cancer in 1990 = 7.1/100 000), Colombia (34.4), Brazil (37.7), Thailand (22.4), and the Philippines (21.6) were selected for the IARC case-control studies to evaluate the role of males in cervical cancer. Overall, 1921 couples were recruited.

The objective was to determine the relationship between men's sexual behaviour and penile HPV, and subsequently, levels of cervical HPV infection and cervical cancer in their partners.

There were some key differences in the sexual life and behaviour patterns in the countries chosen. Men from South America generally had more lifetime sexual partners than men from Thailand, Spain, and the Philippines.

In addition, men from Brazil and particularly Colombia were more likely than those from Spain, Thailand, and the Philippines to have regular intercourse before they were 18 years of age.

#### HPV prevalence

Men from Brazil and Colombia were far more likely to be HPV-positive in the distal urethra than men from Thailand, the Philippines, and Spain. However, this did not necessarily correlate with HPV positivity in their women partners (Table 8).

The ASRs of cervical cancer within the different populations correlated closely with both the prevalence of penile HPV and with cervical HPV infections.

The data clearly demonstrate that in countries with low prevalence of HPV, such as Spain, the risk of cervical cancer in women is influenced by the partner's sexual proclivity [167]. However, in Colombia, where the prevalence of HPV is higher, this is less visible in epidemiological studies, even when some of the male's partners may be prostitutes [168].

The interpretation that can be drawn is as follows.

- HIGH-RISK countries: HPV prevalence in young males is very high and any given sexual contact conveys a high risk of exposure. When risk of infection at any given episode of intercourse is high, increasing the number of sexual partners does not greatly increase this already high risk of infection.
- LOW-RISK countries: HPV prevalence in males is low and only sexual intercourse with high-risk males or a high number of partners for women conveys a high risk of HPV exposure.

#### Circumcision and HPV

In this study, it was more common for men to be uncircumcised (*n* = 847) than circumcised (*n* = 292). Uncircumcised men in the study were > 3 times more likely to be positive for penile HPV (19.6%) than circumcised men (5.5%) [169]. Overall the wives of circumcised men were less likely to develop cervical cancer (OR = 0.75). The relative risk for cervical cancer in monogamous women with circumcised husbands varied with the men's sexual proclivity. A wife whose husband's sexual behaviour was viewed as high risk (> 6 partners and first sexual encounter before 16 years of age) was 0.18 times more likely to suffer cervical cancer; women whose husbands had low-risk sexual behaviour gained no benefit from his circumcision.

#### HPV and population dynamics

In future, public health will need to consider the role of the male in planning management programmes for cervical cancer. It is evident that the risks for both infection and cancer development are influenced not only by the types and frequency of HPV within a population, but also by sexual behaviour. Women who have their first stable sexual relationship at a young age (< 20 years of age) have a greater risk of cervical cancer.

Societal factors play a major role in sexual relationships, and in the case of cervical cancer strongly influence national epidemiology of HPV-associated disease.

#### Condoms, HPV, and cervical dysplasia

Condom use has been associated not only with lower penile infection rates and regression of penile lesions, but also with CIN regression and clearance of cervical HPV [170]. In a 2-year trial of women with CIN lesions (*n* = 82) regression occurred in 53% of those whose partners wore condoms, compared with 35% of those whose partners did not. Among those with condom-wearing partners, 23% cleared the HPV infection, compared with only 4% of those whose partners did not wear condoms.

#### The male contribution to cervical cancer prevention

Males clearly play a role in HPV prevalence within populations. Cervical cancer rates in women would be aided by any of the following:

- abstinence/monogamy/low promiscuity,
- late sexual debut (particularly in women),
- avoiding high-risk partners,
- consistent use of condoms,
- male circumcision,
- vaccination against HPV.

## ANOGENITAL HPV INFECTION AND DISEASE IN HIV-POSITIVE WOMEN

### Isabelle Heard

#### HIV prevalence in Asian adults

HIV infection is not uncommon in many Asian countries. At the end of 2003, Cambodia, Thailand, Myanmar, India, Nepal, and Papua New Guinea had known prevalence levels > 0.5% [171]. As patients continue to survive longer following HIV infection, due to advances in treatment, health practitioners must consider the implications of anogenital HPV infection in HIV-positive women.

#### HPV prevalence in HIV-positive women

A study of women in four US cities found that 60–70% of HIV-positive women tested positive for HPV, making them 2.3 times more likely to be infected than HIV-negative women. They were also 1.9 times more likely to be infected with multiple HPV strains, and 1.6 times more likely to have a higher HPV viral load [172].

Women with HIV were also more likely to have persistent infections with HPV; immunosuppression appears to play an important role in modulating the natural history of HPV infections [173].

Palefsky et al looked specifically at HR-HPV types in HIV-positive women, finding a prevalence of 20–34% with an OR of 5.1 compared with HIV-negative women [174].

Immunosuppression in HIV is monitored through the CD4 count. A lower T-cell count is closely related to likelihood of infection with HPV, including high-risk types, a high viral load, and persistence of the infection [172–174].

#### Cervical disease

A number of studies have demonstrated that the prevalence of cervical cellular abnormalities is significantly higher in HIV-positive women [175–179].

Most cytological abnormalities are ASCUS and low-grade SIL, with high-grade lesions diagnosed in less than 10%.

Again, risk of cervical disease is greater when HPV load is high [180] and when immunosuppression is severe [176, 177]. The significance of HIV viral load is less consistent—Massad et al observed no significant effect [177], yet in Duerr et al study the OR was 7.5 [176].

#### Natural history of cervical disease

Infection with HIV increases the incidence of SIL, almost certainly because HIV-positive women are more likely than HIV-negative women to have persistent infections with HR-HPV. A prospective study showed that one in five HIV-positive women with no evidence of cervical disease developed biopsy-confirmed SILs within 3 years [181].

Four studies [175, 177, 178, 182] have attempted to determine whether cervical disease is less likely to regress in HIV-positive women than in those who are not infected. The studies used different measures and endpoints, and so cannot be directly compared; however, studies by Massad and Schuman demonstrated significant HIV-related differences in the likelihood of cervical disease regression.

Three studies [175, 177, 178] have shown significant differences between HIV-positive women and those without HIV, in progression of cervical disease from LSIL to more advanced cervical disease.

#### Impact of HAART

By maintaining CD4 levels, HAART may impact on the natural history of cervical disease in HIV-positive women. However, results of studies are mixed. Women on HAART were found to be more likely to experience regression of HPV-linked disease in three studies [180, 183, 184] while Moore and Chaisson found no effect on CIN prevalence following antiretroviral therapy [185]. In monitoring cervical disease progression, Lillo et al believed it was independent of HIV treatment [186], while Minkoff et al saw a decreased likelihood for disease progression (OR 0.68) in women on HAART [183].

When HIV-positive women are treated surgically for CIN, they are more likely to experience recurrence of disease than HIV-negative women. Cited recurrence rates have ranged from 39% to 56%, regardless of CIN severity or the mode of treatment (excision versus ablation). The prime factor influencing rates of recurrence is the level of immunodeficiency. Recurrence is halved in women on HAART. The conclusion was that although surgery is highly effective for immunocompetent patients, it only prevents progression to cancer in HIV-positive women [187]. Disease recurrence should be anticipated in immunocompromised patients.

#### Invasive cervical carcinoma

Case reports of invasive cervical cancers in patients with AIDS led the CDC to include cervical cancer as an AIDS-defining illness in 1993 [188, 189].

The risk of ICC among HIV-positive women means that more frequent testing is justified. In a prospective preventative study, six-monthly testing was undertaken in women with CIN1 lesions. These infrequently progressed in women with HIV, so observation appears safe, in the absence of other indications for treatment [190]. In a Swiss study, HIV-positive women had a higher level of cervical cancer than HIV-negative women (standardised incidence ratio [SIR] = 8.0); however, unlike some other AIDS-related cancers (Kaposi sarcoma, non-Hodgkins lymphoma), HAART did not have a statistically significant impact on ICC incidence [191].

#### HPV-related vulvo-vaginal disease

Women with HIV are more likely to experience VIN than HIV-negative women (4.67 versus 1.31/100 person years) [190]. Incident VIN is more common among women who have cervical lesions.

HIV-positive women are also at a higher risk for both invasive and in situ vulvar cancers (relative risk = 5.8 versus 3.9) [192]. Younger women (< 30 years of age) appear to have an even higher risk.

#### Anal disease

Anal HPV infection is frequent (76%) in HIV-positive women [193]. Prevalence of AIN is also high (26%), particularly when immunodeficiency is severe (CD4 < 200 mm^3^) and cervical disease is also present [194]. A European study also reported the high risk of anal cancer among HIV-positive women (SIR = 18.5) [191].

#### Screening tests in HIV-positive women

Because the Pap test has a 10–25% false-negative rate irrespective of HIV status, LBC may provide higher sensitivity than standard cytology. HPV testing may be undertaken at the time of the swab, and should certainly be used to review ASCUS samples.

In HIV-positive women, more frequent screening is recommended if HPV infection is known, there has been a previous abnormal smear, if CD4 < 200, or following any surgical treatment for cervical lesions.

#### Managing HPV-related anogenital lesions

Colposcopy should be used routinely in all HIV-positive women with a known HIV-positive status, if immunosuppressed (CD4 < 200) and following an abnormal Pap smear [195]. The entire lower genital tract should be reviewed and biopsies taken. Following surgical treatment for cervical lesions, four-monthly colposcopy is recommended.

Because colposcopy is a subjective science and prone to false-negatives and -positives, biopsies should be considered early on in the evolution of any abnormality.

Anal Pap smears have not been adopted in the USA. High-resolution anoscopy can be considered, and any abnormal areas sampled.

Vulvo-vaginal treatments for HPV-related lesions vary in efficacy. Even in immunocompetent patients efficacy ranges are broad (20–80%). The overall response to many treatments is likely to be lower in HIV-positive patients, and recurrence is 3.3 times more frequent.

In the case of AIN, treatment decisions are based on size of the lesion, its location, and grade of histology. In managing these lesions in HIV-positive women, the least aggressive approach is preferred. Radiation therapy should be avoided in the absence of invasive cancer.

Treatment of CIN and AIN should not be modified for patients on HAART, nor should antiretroviral therapy be instituted or modified as part of treatment.

## SINGLE VISIT APPROACH TO CERVICAL CANCER PREVENTION: LESSONS FROM THAILAND

### Khunying Kobchitt Limpaphayom

#### Goals and objectives of cervical screening

The primary goal for Thailand is to reduce cervical cancer and mortality by detecting disease early, and treating it before it progresses to invasive cancer.

Thailand has ∼15 million women at risk for cervical cancer, only 5% of whom have been screened in the previous 5 years via the Pap smear campaign. A major problem is the shortage of trained cytopathologists to examine Pap smears, and limited resources to followup women with positive Pap test results.

#### The single-visit approach

In considering an alternative to conventional cytology, social and logistical in addition to medical challenges need to be met. Key components to be addressed include

- outreach and education—to increase demand and enhance coverage,
- advocacy and policy—to ensure support for the programme,
- training,
- service delivery system—to ensure women have access to screening and treatment that are acceptable to them and sustainable over time,
- a referral system—for women with advanced disease,
- information management—to monitor progress,
- equipment procurement, repair, and maintenance.

A single-visit approach to cervical cancer prevention, linking screening and treatment, is appropriate for low-resource settings. This can be implemented at the lowest level of the health system, can be performed by trained nonphysicians, requires few materials, and is relatively inexpensive. This approach also avoids the delay of sending specimens away for testing.

VIA is now established as a safe, effective, viable alternative to cytology-based screening for low resource settings. Cryotherapy using a CO_2_-based system can be offered to any woman with abnormalities at the time of the screening. There may not be a confirmed pathological diagnosis, but it is the intervention, rather than the diagnosis, that will prevent cervical cancer. Thus, although VIA may not be the most accurate test, it is the most efficient way to provide preventative services to the majority of women at risk.

The major advantage is cost savings. A single visit approach is likely to be more effective and more cost-effective than multiple visit strategies. A once-in-lifetime test for all women could reduce cervical cancer incidence and mortality by up to 25%, dependent on coverage and age groups targeted. Good outcomes have been achieved in settings with a high prevalence of HIV [196–198].

#### The SAFE demonstration project

**Figure 6:**
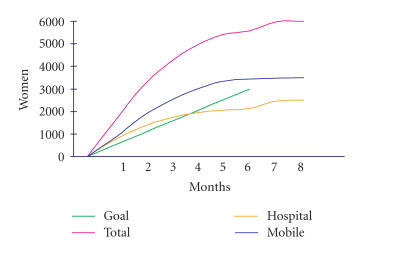
Recruitment—Roi Et trial project, 1999.

In 1999, a trial project based on the single visit approach was initiated in Roi Et province. The safe, acceptability, feasibility (SAFE) project was very successful. Three in four targeted women presented to hospital and mobile clinics in the first 12 weeks, exceeding recruitment goals (Figure 6).

Trained and motivated nurses screened 5999 women over 6 months; 13% were positive with VIA and offered treatment.

Following their experience with SAFE, treated women were surveyed; < 2% felt they were not adequately informed about the treatment, or were not satisfied about their decision to be treated. Most importantly, 96.6% said they would recommend the service to friends and family.

After screening or treatment, 4.9% of women presented again. Only 2.7% of visits concerned a minor complication such as vaginitis, cervicitis, or cramping, and there were no major complications.

Efforts are now focused on training more local nurses in VIA and cryotherapy. By May 2005, ∼ 100 000 women 30–45 years of age had been tested with VIA, and 8–10% were offered treatment with cryotherapy. Ten Thai provinces have now adopted the single visit approach to screening.

#### Keys for success

Four key actions have contributed to the success of the single visit approach in Thailand.

1. Recognition of the importance of linking testing to treatment.
2. Use of competency-based training methods to prepare service providers, clinical supervisors, and master trainers.
3. Use of mobile and static clinics for service delivery, ensuring easier access for women.
4. Communication to build national consensus and support for the programme.

## GLOBAL CERVICAL CANCER CONTROL

### Edward Trimble

#### Global needs for cancer control

Four key areas will contribute to global cancer levels in coming decades:

- oncological infectious agents, that is, HBV, HPV, HIV, Epstein Barr virus, schistosomes, and *Helicobacter pylori*,
- carcinogens, most notably tobacco,
- lifestyle changes, particularly growing obesity and decreased exercise and physical fitness,
- changing age structure in many societies—smaller families and longer life-spans mean that cancer, generally a disease of old age, will grow in prevalence.

#### The global commitment to cancer control

Cancer control is a continuum where improved outcomes can be achieved at many different levels:

- prevention,
- screening/diagnosis,
- treatment,
- symptom management and health-related quality of life leading to prolonged survival,
- end-of-life care.

A number of global organisations are working with national and local groups to develop cancer control programmes that involve stakeholders at all levels, are appropriate to the patient population, and are achievable and sustainable.

Organisations that have publicly stated their commitment to cancer control include the WHO IARC, World Charter Against Cancer 2000, WHO World Health Assembly, and the International Union Against Cancer.

#### GLOW

The Global Initiative on Women's Cancer (GLOW) has identified three areas of focus—gynaecological cancer (especially cervical), breast cancer, and tobacco control.

GLOW supports a number of initiatives for cervical cancer and is developing appropriate guidelines for their adoption:

- prevention with prophylactic HPV vaccines,
- screening,
- new diagnostic tests,
- treatments to cure or prolong survival,
- Palliative care.

Another key focus for GLOW is education through publications, websites, meetings, and satellite symposia at major obstetrics/gynaecology and oncology meetings. GLOW can also provide technical expertise in planning, implementation, and evaluation of cervical control programmes.

The short-term agenda for GLOW includes

- establishing a database for country-specific needs assessment,
- integrating cervical cancer control into national cancer plans and health programmes—including government support and early adoption of HPV vaccination,
- improving availability of treatment and care—by building on existing resources to ensure that cervical cancer screening is part of health promotion for older women.

## Figures and Tables

**Table 1:** Triage test performance of HC II and cytology [11].

	% sensitivity	% referral	Positive predictive value	Negative predictive value

CIN3+
HC II	96.3	56.1	10.0	99.5
HSIL+ cytology	44.1	6.9	37.5	99.5
LSIL+ cytology	64.0	26.2	14.3	97.1
ASCUS+ cytology	85.3	58.6	8.5	97.9
CIN2+
HC II	95.9	56.1	19.6	98.9
HSIL+ cytology	34.8	6.9	58.1	92.0
LSIL+ cytology	59.2	26.2	25.9	93.6
ASCUS+ cytology	85.0	58.6	16.7	95.8

**Table 2:** ^†^Reduction (%) in life years lost according to policy and coverage.

	Netherlands,	Beligum, France	Germany
	Finland	Greece, Italy, Spain	

Starting age	30 years	25 years	20 years
Interval	5 years	3 years	1 year
Ending age	60 years	64 years	72 years
Lifetime no. tests	7	14	53

Interval coverage	% Reduction in life-years lost		

25	21	24	25
50	42	47	50
75	62	71	75
100	84	94	99.9

^†^Adapted from Ballegooijen (2000).

**Table 3:** Comparison between acetic acid visualisation and Pap smears.

		AA^†^	MAA	Smear S	Smear CB

Colposcopy	Sensitivity	50.3%	49.1%	21.0%	17.3%
		(45–56)	(44–54)	(17–25)	(13–22)

	Specificity	94.1%	93.2%	99.1%	98.6%
		(93–95)	(92–94)	(99–99)	(98–99)

Biopsy	Sensitivity	37.1%	34.1%	14.6%	19.5%
		(28–46)	(26–42)	(14–19)	(10–29)

	Specificity	92.6%	90.2%	98.2%	98.3%
		(92–94)	(90–93)	(98–99)	(98–99)

^†^Other studies: AA = 51–82%; PS = 13–85% snr.

**Table 4:** Accuracy of screening tests for CIN 2+ compared with pathology.

Screening test		Accuracy in detecting lesions	
		(versus pathology)	

		SPOCCS I	SPOCCS II
		(*n* = 1997)	(*n* = 8497)

HPV self-test		85.8%	81.4%
HPV direct test		85.4%	84.0%

Pap-LBC	ThinPrep	93.2%	—
	Autocyte	—	81.7%

Colposcopy		76.7%	92.5%
Visual inspection		74.2%	89.1%
Fluorescent spectroscopy		0.1%	—

**Table 5:** Contribution of HPV types 6, 11, 16, and 18 to HPV-related disease.

HPV Type	Women	Men

6/11	90% of genital warts [65, 66]	90% of genital warts [65, 66]
	5–25% of low-grade cervical lesions	Transmission to women

16/18	25% of low-grade cervical lesions	70% of AIN [66, 67] 70% of anal cancer Transmission to women
	70% of high-grade cervical lesions	
	70% of cervical cancer [67]	
	70% of other genital cancers	

**Table 6:** Coding systems used in reviewing recommendations.

Strength of recommendations	Quality of evidence

(A) *Good evidence* for efficacy and substantial clinical benefit supports recommendation for use	(I) Evidence from at least one randomised controlled trial
(B) *Moderate evidence* for efficacy or only limited clinical benefit supports recommendation for use	

(C) *Evidence* of efficacy is *insufficient* to support a recommendation for or against use, but recommendation may be made on other grounds	(II) Evidence from at least one clinical trial without randomisation, from cohort or case-controlled analytic studies, or from multiple time series studies or dramatic results from uncontrolled experiments
(D) *Moderate evidence* for *lack of efficacy* or for adverse outcome supports a recommendation against use	

(E) *Good evidence* for *lack of efficacy* or for adverse outcome supports a recommendation against use	(III) Evidence from opinions of respected authorities based on clinical experience, descriptive studies, or reports of expert committees

**Table 7:** Impact of screening interval on incidence of disease.

Screening interval (years)	Reduction in incidence^†^ (%)	No. of cervical during lifetime smears

1	93%	45
3	91%	15
5	84%	9
1 smear at 40 years	20%	1

^†^Assuming 100% coverage for women 20–64 years of age.

**Table 8:** Cervical and penile HPV prevalence in control couples.

	% HPV-positive	
	Men	Women

Brazil	29.7	17.3
Colombia	18.9	15.3
Thailand	9.2	15.7
Philippines	4.7	9.2
Spain	3.5	5.4
